# Wheat yellow mosaic virus NIb targets TaVTC2 to elicit broad‐spectrum pathogen resistance in wheat

**DOI:** 10.1111/pbi.14019

**Published:** 2023-02-14

**Authors:** Tianye Zhang, Haichao Hu, Ziqiong Wang, Tianyou Feng, Lu Yu, Jie Zhang, Wenqing Gao, Yilin Zhou, Meihao Sun, Peng Liu, Kaili Zhong, ZhiHui Chen, Jianping Chen, Wei Li, Jian Yang

**Affiliations:** ^1^ State Key Laboratory for Quality and Safety of Agro‐products, Institute of Plant Virology Ningbo University Ningbo China; ^2^ Guizhou University Guiyang Guizhou China; ^3^ State Key Laboratory of Plant Genomics, Institute of Microbiology Chinese Academy of Sciences Beijing China; ^4^ State Key Laboratory for Biology of Plant Diseases and Insect Pests, Institute of Plant Protection Chinese Academy of Agricultural Sciences Beijing China; ^5^ College of Chemistry and Life Science Zhejiang Normal University Jinhua China; ^6^ School of Life Sciences University of Dundee Dundee UK; ^7^ Hunan Provincial Key Laboratory for Biology and Control of Plant Diseases and Insect Pests, College of Plant Protection Hunan Agricultural University Changsha China

**Keywords:** wheat, TaVTC2, ascorbate acid, reactive oxygen species, NIb, wheat yellow mosaic virus

## Abstract

GDP‐L‐galactose phosphorylase (*VTC2*) catalyses the conversion of GDP‐L‐galactose to L‐galactose‐1‐P, a vital step of ascorbic acid (AsA) biosynthesis in plants. AsA is well known for its function in the amelioration of oxidative stress caused by most pathogen infection, but its function against viral infection remains unclear. Here, we have identified a *VTC2* gene in wheat named as *TaVTC2* and investigated its function in association with the wheat yellow mosaic virus (WYMV) infection. Our results showed that overexpression of *TaVTC2* significantly increased viral accumulation, whereas knocking down *TaVTC2* inhibited the viral infection in wheat, suggesting a positive regulation on viral infection by *TaVTC2*. Moreover, less AsA was produced in *TaVTC2* knocking down plants (*TaVTC2*‐RNAi) which due to the reduction in *TaVTC2* expression and subsequently in TaVTC2 activity, resulting in a reactive oxygen species (ROS) burst in leaves. Furthermore, the enhanced WYMV resistance in *TaVTC2*‐RNAi plants was diminished by exogenously applied AsA. We further demonstrated that WYMV NIb directly bound to TaVTC2 and inhibited TaVTC2 enzymatic activity in vitro. The effect of TaVTC2 on ROS scavenge was suppressed by NIb in a dosage‐dependent manner, indicating the ROS scavenging was highly regulated by the interaction of TaVTC2 with NIb. Furthermore, *TaVTC2* RNAi plants conferred broad‐spectrum disease resistance. Therefore, the data indicate that TaVTC2 recruits WYMV NIb to down‐regulate its own enzymatic activity, reducing AsA accumulation to elicit a burst of ROS which confers the resistance to WYMV infection. Thus, a new mechanism of the formation of plant innate immunity was proposed.

## Introduction

Ascorbate acid (AsA) is a primary water‐soluble antioxidant both in plants and animals (De Tullio and Arrigoni, 2004). It acts either as a direct scavenger of ROS and indirectly as a substrate for ascorbate peroxidase (APX) in the neutralization of H_2_O_2_ (Jimenez *et al*., 1997). When scavenging ROS, AsA can be oxidized by APX to yield monodehydroascorbate (MDHA). Then MDHA is further hydrolysed to generate dehydroascorbate (DHA). MDHA and DHA can be reconverted to AsA in the AsA recycling pathway catalysed by MDHA reductase (MDHAR) and DHA reductase (DHAR) respectively (Smirnoff and Wheeler, 2000). However, the role of AsA in plant resistance to biotic stress such as pathogen infection is still undefined.

Four pathways can be used for AsA biosynthesis in plants including the L‐galactose, L‐gulose, myo‐inositol, and D‐galacturonate pathways, of which the L‐galactose pathway is the dominant pathway (Linster and Clarke, 2008). GDP‐L‐galactose phosphorylase (VTC2) catalyses the conversion of GDP‐L‐galactose to L‐galactose‐1‐P, which is the first and vital step of AsA biosynthesis (Bulley *et al*., 2009; Laing *et al*., 2007; Linster *et al*., 2007). Studies showed that overexpression of *VTC2* enhancing the AsA level in plants then improves multi‐stress tolerance including salt stress, chilling stress, and oxidative stress (Luo *et al*., 2021; Vidal‐Meireles *et al*., 2017; Wang *et al*., 2014). In contrast, the *Arabidopsis vtc2* mutant has low VTC2 activity but shows more resistance to plant pathogens, such as *Pseudomonas syringae* and *Peronospora parasitica*, than the wild type (Barth *et al*., 2004), partly due to the increase in salicylic acid (SA) level in the *Arabidopsis vtc2* mutant, which induced the expression of antimicrobial pathogenesis‐related (PR) proteins (Mukherjee *et al*., 2010; Pavet *et al*., 2005). Another reason could be that the lower VTC2 activity and consequently the lower AsA content will result in a ROS burst, which confers the resistance to many pathogens.

It has been suggested that a local ROS burst can limit virus spread and ROS also act as signalling molecules to induce or trigger antiviral immunity, such as the hypersensitive reaction (HR) and systemic acquired resistance (SAR) (Durrant and Dong, 2004; Li *et al*., 2021; Mittler, 2017). For instance, the citrus tristeza virus (CTV) p33 protein activates the ROS‐mediated host immune response to restrict CTV to the phloem tissue and inhibit viral infection (Sun and Folimonova, 2019). Moreover, it was demonstrated that the promotion of ROS accumulation with the microRNA528 could activate antiviral immunity in rice (Wu *et al*., 2017). On the other hand, viruses could enhance ROS production so that ROS is used to initiate and accelerate viral infection. The Red clover necrotic mosaic virus, RCNMV p27 protein utilizes a respiratory burst oxidase homologue (RBOH) of *N. benthamiana* to induce an intracellular ROS burst for robust viral RNA replication (Hyodo *et al*., 2017). It is also the case that the helper component proteinase (HCPro) of chilli veinal mottle virus interacts with *Nicotiana tabacum* catalase, which induces a ROS burst to facilitate viral infection (Yang *et al*., 2020b). Therefore, ROS may play different roles during different viral infection.

WYMV is a positive‐sense RNA virus that belongs to the genus *Bymovirus* in the *Potyviridae* family. WYMV NIb protein functions as an RNA‐dependent RNA polymerase (Chen *et al*., 2014). Previous studies have shown that the NIb of *Potyviruses* plays a role in multiple functions during viral infection (Li *et al*., 2018a; Shen *et al*., 2020). For example, NIb is not only a good recruiter that coopts many host proteins like poly(A)‐binding protein (Wang *et al*., 2000) and RNA helicase‐like protein (Huang *et al*., 2010) to accelerate viral infection but also can act as a suppressor of host defence responses to promote viral infection (Shen *et al*., 2020). TuMV NIb competes against NPR1 for and/or depletes SUMO3 to suppress SUMO3‐activated NPR1‐mediated immune response (Cheng *et al*., 2017). Furthermore, NIb is thought to be an ETI elicitor that induces ROS accumulation and triggers host resistance to inhibit viral infection (Kim *et al*., 2018). Therefore, the multifunction of potyviral NIb protein remains to be further explored.

In this study, we first identified a *VTC2* gene in the wheat genome and then investigated its function in antiviral immunity in relation to AsA production and ROS scavenge by overexpressing or RNAi knocking down this gene. We found this gene could positively regulate the WYMV infection. Then, we investigated the mechanism of such a positive regulation by screening protein interactions between the host wheat and the WYMV. We found that a WYMV protein NIb physically interacts with TaVTC2 and inhibits TaVTC2 catalytic activity for AsA production in vivo and in vitro. We also tested the effect of exogenously applied AsA on the decreased susceptibility to WYMV infection in the *TaVTC2*‐RNAi plants. Finally, we proposed a new model of the innate immunity formation in plants.

## Results

### TaVTC2 positively regulates WYMV infection in wheat

**Figure 1:**
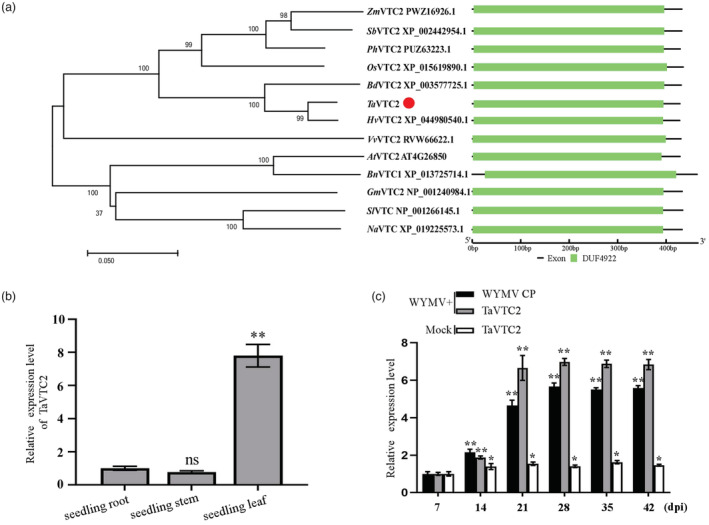
Identification of TaVTC2 protein and expression pattern of *TaVTC2* in wheat plants. (a) Phylogenetic and conserved domain analysis of VTC2 from different species. The VTC2 protein sequences were obtained from *Sorghum bicolor* (Sb), *Zea mays* (Zm), *Setaria italica* (Si), *Panicum hallii* (Ph), *Brachypodium distachyon* (Bd), *Hordeum vulgare* (Hv), *T. aestivum* (Ta), *Oryza sativa* (Os), *Arabidopsis thaliana* (At), *Brassica napus* (Bn), *Solanum lycopersicum* (Sl), *Vitis vinifera* (Vv), *Nicotiana attenuata* (Na), *Sorghum bicolor* (Sb), and *Glycine max* (Gm). TaVTC2 is highlighted in the phylogenetic tree using a red circle. The phylogenetic tree was constructed by the neighbour‐joining method in MEGA 7.0 software, with bootstrap values of 1000. The percentage on phylogenetic tree nodes indicated that the associated taxa clustered together in the bootstrap test. The branch lengths represent evolutionary distances, which are calculated using p‐distance method. (b) Expression of *TaVTC2* in different tissues of wheat plants. (c) Expression of *TaVTC2* and accumulations of WYMV in WYMV‐inoculated or mock‐inoculated wheat plants. The expression level of the *TaActin* gene was used as an internal control. Each relative expression level is presented as mean ± SD from three biological triplicates. Statistical analysis was performed using Student's *t*‐test. *P < 0.05; **P < 0.01; ns: no significant difference.

We first identified a VTC2 protein in the wheat genome by BlastP using Arabidopsis VTC2 (AtVTC2), which was further confirmed by cloning and sequencing a full‐length cDNA of this gene. Then, we constructed a phylogenetic tree and analysed the conserved domains in this new protein sequence and homologues from other species. The results showed that this *VTC2* shares a close relationship with *Hordeum vulgare* VTC2 (*Hv*VTC2), *Brachypodium distachyon* VTC2 (*Bd*VTC2), *Oryza sativa* VTC2 (*Os*VTC2), and *Arabidopsis* VTC2 (*At*VTC2) (Figure 1a). Thus, we named this gene as *TaVTC2* and deposited it in the NCBI database (GenBank accession no. MK514263.1). This gene was expressed mostly in leaves of the wheat seedlings (Figure 1b) and its expression was responsive to WYMV infection as its expression level increased following the increasing loading of virus indicated by the WYMV CP expression (Figure 1c), suggesting that *TaVTC2* may be involved in WYMV infection.

**Figure 2:**
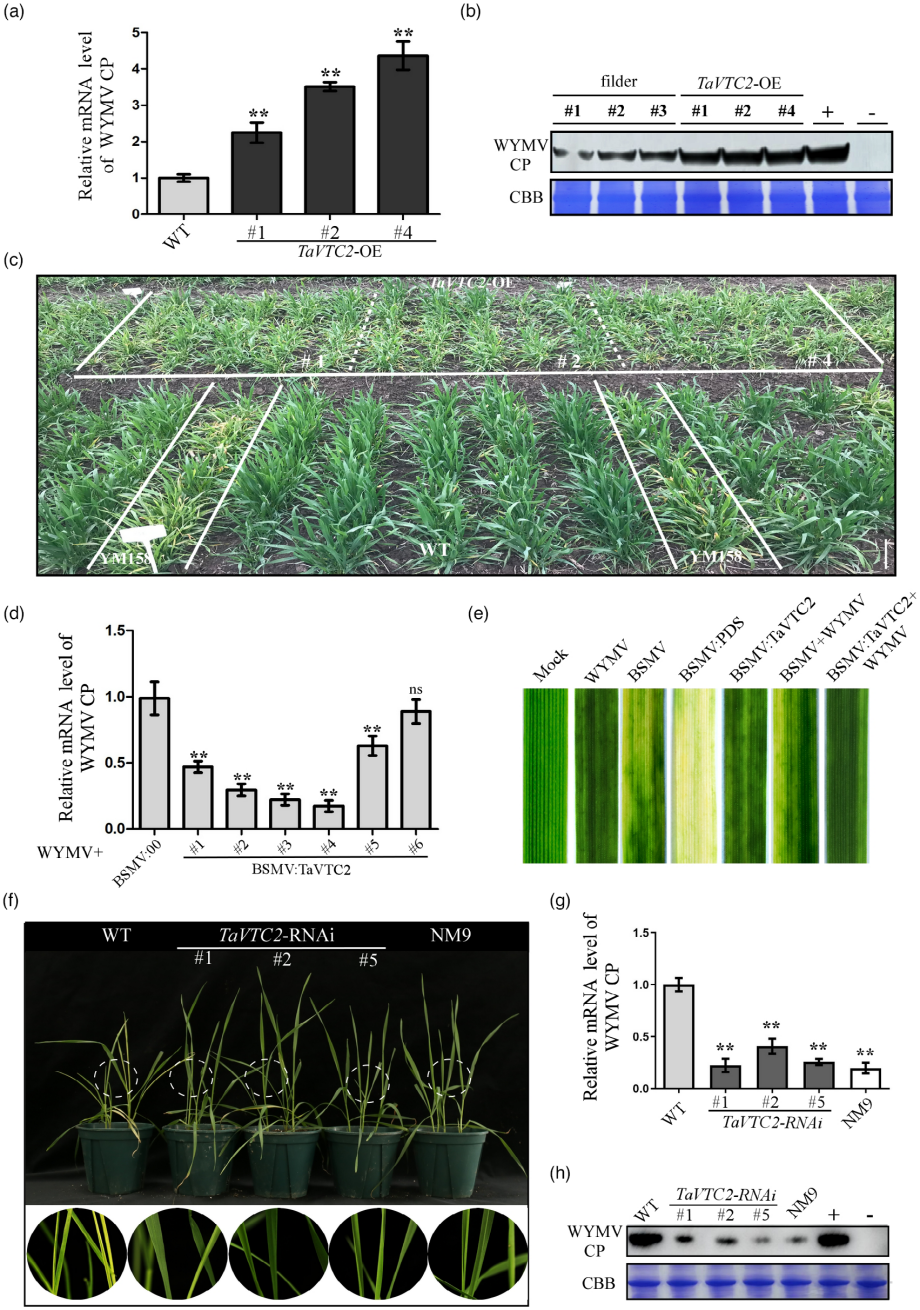
TaVTC2 positively regulates WYMV infection. (a) WYMV accumulation in the *TaVTC2*‐OE and WT plants in field were determined by qRT‐PCR. # 1,2,4 represent three independent lines of *TaVTC2*‐OE. (b) Detection of WYMV infection in plants by Western blot using a WYMV CP‐specific antibody. (c) Assessment of *TaVTC2*‐OE plants for disease resistance in a virus‐contaminated nursery at Yangzhou, Jiangsu Province in 2020. YM158, which is susceptible to WYMV, was used as the negative control. WT represents fielder plants. (d) Relative expression levels of WYMV CP in six wheat plants co‐infected with WYMV and BSMV: TaVTC2. Total RNA from BSMV and CWMV co‐infected plants (BSMV:00) were used as negative control. (e) Phenotypes in the fourth leaves of the plants inoculated with phosphate‐buffered saline (Mock), WYMV, BSMV, BSMV:PDS, BSMV:TaVTC2, BSMV+CWMV and BSMV:TaVTC2+WYMV respectively. Photographs were taken after 14 days post‐inoculation (dpi) with virus. (f) Phenotypes after 14 dpi of *TaVTC2*‐RNAi plants inoculated with WYMV. The white dotted circle represents the area magnified below. # 1,2,5 represent three independent lines of *TaVTC2*‐RNAi. WT represents YM158 plants. (g, h) The protein level or mRNA level of WYMV accumulation in the *TaVTC2*‐RNAi and WT plants were determined by qRT‐PCR and Western blot respectively. Coomassie brilliant blue (CBB)‐stained bands of rubisco large subunit show equal protein loadings in each lane. Each relative expression level is presented as mean ± SD from three biological triplicates. Statistical analyses were done using Student's *t*‐test. *P < 0.05; **P < 0.01; ns: no significant difference.

To investigate the function of *TaVTC2* associated to WYMV infection, the overexpression of *TaVTC2* (*TaVTC2*‐OE) was confirmed in T0 transgenic ‘Fielder’ wheat (a wild‐type *T. aestivum* variety with resistance to WYMV) lines by a Western blot with anti‐Flag antibodies (Figure S1a) and tested again in T2 lines derived from T1 lines (Figure S1b). ddPCR revealed that these transgenic lines all contained a single copy of *TaVTC2* (Figure S1c–f). Next, T3 transgenic plants *TaVTC2*‐OE were inoculated with an RNA transcript of WYMV. After 7 dpi, we detected WYMV infection by the accumulation of WYMV CP, measured by qRT‐PCR in these inoculated seedlings. The results showed that WYMV CP accumulation in *TaVTC2*‐OE lines was significantly higher than in ‘Fielder’ WT plants (Figure 2a), which was also confirmed by the Western blot analysis (Figure 2b). We also observed that the mosaic symptoms in WYMV‐inoculated *TaVTC2*‐OE plants were more severe than in WYMV‐inoculated WT plants (Figure 2c). In addition, the agronomic performance of these three T3 transgenic lines in the field tests did not show any obvious difference to that of ‘Fielder’ and YM158 plants when grown in a virus‐free nursery. However, when grown in a virus‐contaminated nursery, the spike length and seed quality of these *TaVTC2*‐OE transgenic lines were inferior to those of ‘Fielder’ plants (Figure S2a–c). The above results clearly demonstrated that the *TaVTC2*‐OE plants were more susceptible to WYMV infection (Figure 2a–c), suggesting that TaVTC2 might positively regulate the WYMV infection.

To further analyse the effects of *TaVTC2* on WYMV infection, we tried to generate a *TaVTC2*‐knockout mutant, but without success. Therefore, the Barley stripe mosaic virus (BSMV) was applied to silence or knock‐down *TaVTC2*. First, we constructed two recombinant plasmids, BSMV:TaVTC2 and BSMV:TaPDS (phytoene desaturase gene), then we inoculated wheat seedlings with WYMV, BSMV, BSMV:TaPDS (which acted as a positive control), BSMV:TaVTC2, BSMV+WYMV, or BSMV:TaVTC2+WYMV. After 14 dpi, the seedlings infected with WYMV and BSMV, respectively, were evaluated by RT‐PCR to detect for the expression of WYMV CP gene or the BSMV CP gene (Figure S3a). As shown in Figure S3b, the transcription of *TaVTC2* in wheat seedlings inoculated with BSMV:TaVTC2+WYMV was significantly silenced. Furthermore, WYMV CP expression in seedlings inoculated with BSMV:TaVTC2+WYMV was significantly lower than the control with BSMV:00+WYMV (Figure 2d). In addition, at 14 dpi, systemically infected leaves of wheat seedlings inoculated with BSMV:TaPDS exhibited a typical photobleaching phenotype, whereas the leaves of plants inoculated with BSMV:TaVTC2+WYMV displayed milder mosaic symptoms compared to that of plants inoculated with BSMV or BSMV+WYMV (Figure 2e). Furthermore, we generated transgenic *TaVTC2*‐RNAi plants on background YM158 (a wild‐type *T. aestivum* variety susceptible to WYMV) by transformation to express hairpin RNAs with a segment of the conserved region of *TaVTC2*. As shown in Figure S3c, *TaVTC2* was silenced in *TaVTC2*‐RNAi plants. Next, *TaVTC2*‐RNAi plants as well as YM158 and NM9 (a wild‐type *T. aestivum* variety resistant to WYMV) were inoculated with WYMV. After 21 dpi, the systemically infected leaves of *TaVTC2*‐RNAi plants exhibited much less mosaic symptoms than in the inoculated leaves of the YM158 plants, but similar mosaic symptoms were seen in the NM9 plants (Figure 2f). The mRNA and protein accumulation of WYMV CP detected by qRT‐PCR and Western blot were more significantly decreased in *TaVTC2*‐RNAi lines than in YM158 plants when both inoculated with WYMV (Figure 2g,h), indicating that the *TaVTC2*‐RNAi plants were more resistant to WYMV infection. Therefore, the *TaVTC2*‐RNAi data strongly support the above conclusion drawn from the *TaVTC2*‐OE plants that *TaVTC2* positively regulated the WYMV infection.

### TaVTC2 interacts with WYMV NIb in vivo and in vitro

**Figure 3:**
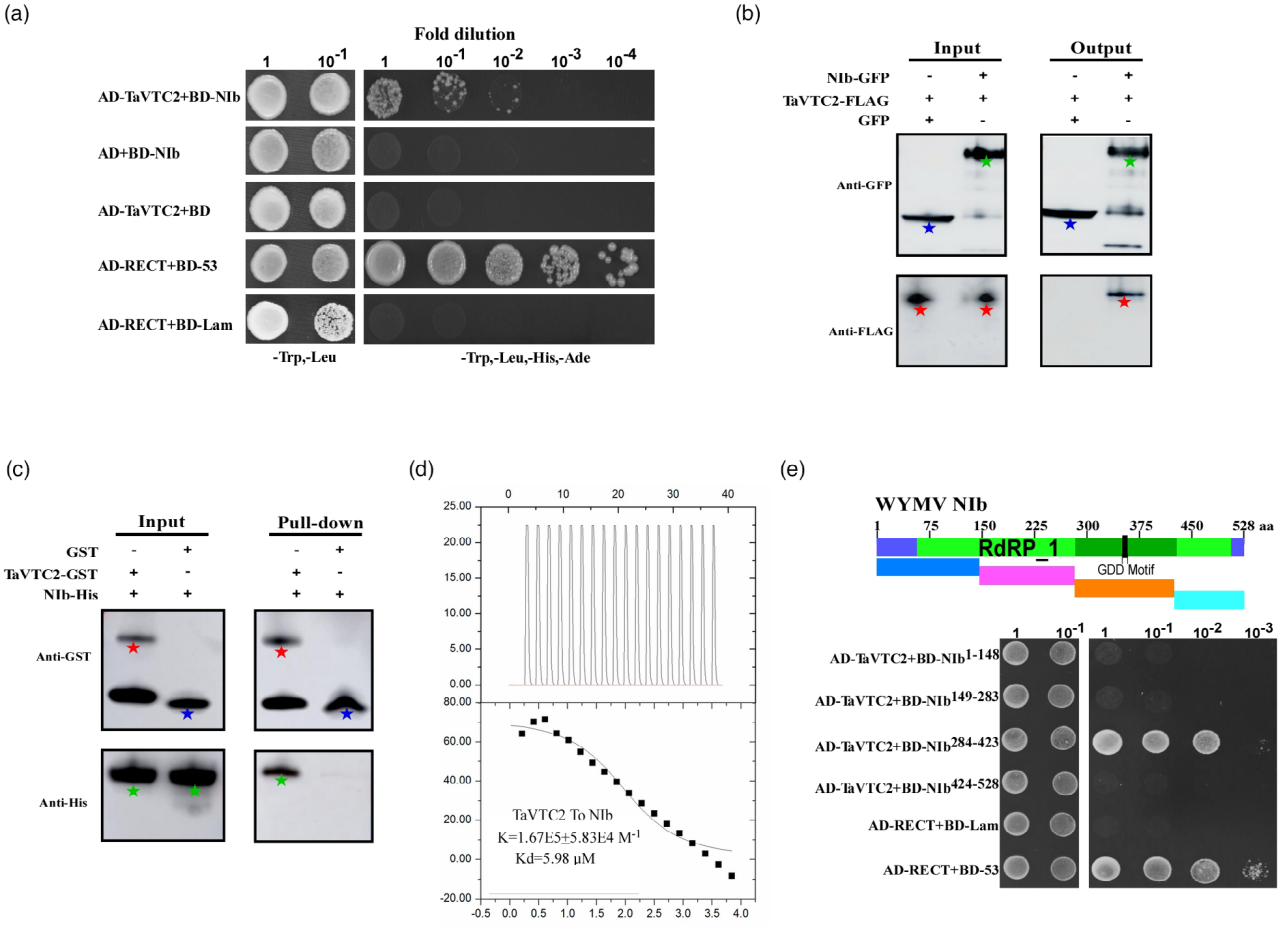
TaVTC2 interacts with NIb. (a) Yeast‐two hybrid (Y2H) assay for interaction between TaVTC2 and NIb. The positive and negative controls are yeast cells co‐transformed with pGAD‐T plus pGBK‐53 and pGAD‐T plus pGBK‐Lam respectively. (b) Co‐IP analysis of the association of NIb with TaVTC2 *in planta*. NIb‐GFP proteins together with TaVTC2‐Flag were co‐expressed in *N. benthamiana* leaves and harvested at 3 dpi. Total proteins were IP with anti‐GFP beads. Total and IP products were detected in Western blots with anti‐GFP or anti‐Flag antibodies. Green pentacle: NIb‐GFP; red pentacle: TaVTC2‐Flag; blue pentacle: GFP. (c) GST pull‐down assays are used to evaluate in vitro interaction between TaVTC2 and NIb. Purified NIb‐His protein was incubated with TaVTC2‐GST and GST protein respectively. Input and pull‐down products were analysed by Western blots with anti‐GST or anti‐His antibodies. Green pentacle: NIb‐His; red pentacle: TaVTC2‐GST; blue pentacle: GST. (d) ITC results of recombinant TaVTC2‐GST and recombinant Nib‐His (*K* = 1.67E5 ± 5.83E4 M^−1^). (e) Y2H assays to detect possible interactions between NIb‐truncated proteins (NIb^1–148^, NIb^149–283^, NIb^284–423^, and NIb^424–528^) and TaVTC2 protein.

Successful viral infection is dependent on the complicated interaction network between the virus and host factors in plants. To understand the molecular mechanism involved in the regulation of WYMV infection by TaVTC2, we performed a yeast two‐hybrid (Y2H) assay using TaVTC2 as bait to screen a WYMV cDNA library. Sequencing of positive clones showed that the WYMV NIb was the top hit. Subsequently, an Y2H assay with TaVTC2 bait and NIb target was performed to confirm their interactions as shown in Figure 3a, indicating a strong interaction between TaVTC2 and WYMV NIb in yeast cells. This interaction was further confirmed by a co‐immunoprecipitation (Co‐IP) assay from the *N. benthamiana* tabaco cells co‐expressed with NIb‐GFP and TaVTC2‐Flag driven by the 35S promoter. Clearly, the GFP antibody could specifically pull‐down both NIb‐GFP and TaVTC2‐Flag (Figure 3b). In order to further examine whether TaVTC2 physically interacts with NIb, we purified both TaVTC2‐GST and NIb‐His that expressed in *E. coli*. BL21, respectively, and performed a GST pull‐down in vitro assay. The results showed that TaVTC2‐GST could pull‐down NIB‐His, indicating a direct interaction between TaVTC2 and NIb (Figure 3c). Additionally, isothermal titration calorimetry (ITC) analysis also revealed that TaVTC2‐GST bound to NIb‐His with a K value 1.67E5 ± 5.83E4 M^−1^ which indicated an affinity at micromolar levels between TaVTC2 and NIb (Figure 3d). Furthermore, to identify the key domain of WYMV NIb for interaction, the full‐length NIb sequence containing 528 amino acid was divided into four fragments, NIb^1–148^, NIb^149–283^, NIb^284–423^, and NIb^424–528^. Of these four fragments, only the NIb^284–423^ fragment interacted strongly with TaVTC2 in yeast (Figure 3e). Altogether, these results indicated that TaVTC2 can bind NIb both in vitro and in vivo, and the NIb C‐terminal (284–423 amino acid) is essential for such an interaction.

### NIb suppresses TaVTC2 enzyme activity to interfere its binding with substrate

**Figure 4:**
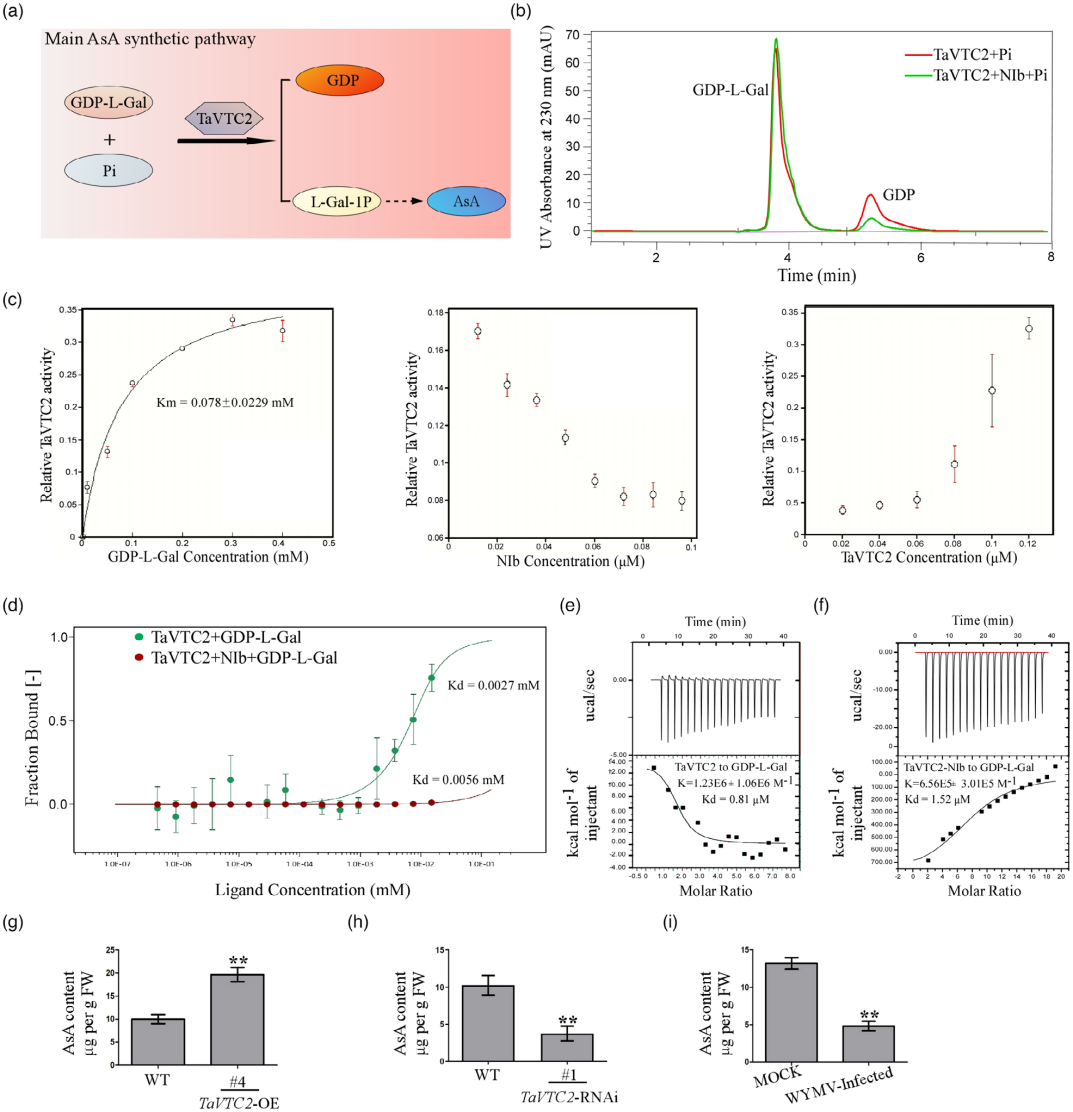
WYMV NIb inhibits TaVTC2 enzyme activity. (a) A brief model of AsA synthetic pathways in plants. (b) TaVTC2 activity was assayed by phosphate‐dependent production of GDP from GDP‐L‐galactose. The GDP in two groups was determined via HPLC at 230 nm absorbance. GDP‐L‐Gal: GDP‐L‐galactose. (c) Enzyme kinetic analysis of TaVTC2. Right: Dependence of TaVTC2 activity on GDP‐L‐Gal concentration. The *K*_m_ value, calculated by fitting the data to the Michaelis–Menten equation, is 0.078 ± 0.0229 mm. Mid: Changes of TaVTC2 activity with the increasing of NIb content. Left: TaVTC2 activity assayed by varying the TaVTC2 concentration in presence of constant concentration of NIb. The error bars represent the SD of each data point calculated from three independent measurements. (d) MST measurements of TaVTC2+GDP‐L‐Gal (*K*_d_ = 0.0027 mm) and TaVTC2‐NIb+GDP‐L‐Gal (*K*_d_ = 0.0056 mm). (e, f) ITC results of TaVTC2+GDP‐L‐Gal (*K* = 1.23 ± 1.06 μm) and TaVTC2‐NIb+GDP‐L‐Gal (*K* = 0.656 ± 0.3 μm). (g–i) Measurement of AsA content in WT (Fielder)/*TaVTC2*‐OE, WT (YM158)/*TaVTC2*‐ RNAi, and MOCK‐inoculated/WYMV‐infected. Each data (mean ± SD) was from at least three biological triplicates. Statistical analyses were done using Student's *t*‐test. *P < 0.05; **P < 0.01.

TaVTC2 is a rate‐limiting enzyme of the AsA synthetic pathway in plants (Linster and Clarke, 2008). Here, we further investigated whether the interaction of TaVTC2 with NIb affect the activity of TaVTC2 in plants. The catalytic substrate (GDP‐L‐galactose, GDP‐L‐Gal) of TaVTC2 was synthesized and both NIb‐His and TaVTC2‐GST fusion protein were purified in vitro. Subsequently, we tested the effect of NIb protein on the enzyme activity of TaVTC2 in vitro through measurement of its substrates and products in the reaction: GDP‐L‐galactose + Pi → L‐galactose 1‐P + GDP (Figure 4a) (Linster *et al*., 2007) *via* high‐performance liquid chromatography (HPLC). The data showed that the production of GDP was reduced in the presence of NIb protein (Figure 4b). Subsequently, we undertook a TaVTC2 enzyme kinetics assay and found a *K*_m_ value of 0.078 ± 0.0229 mm for GDP‐L‐Gal (Figure 4c). Interestingly, when TaVTC2 enzyme concentration was kept constant at 0.05 μm in the reactions and gradually increasing the content of NIb, the catalytic activity of TaVTC2 gradually decreased following the increase in NIb and reached almost the bottom when the content of NIb increased over 0.06 μm (Figure 4c). Similarly, when NIb concentration was kept at a constant 0.06 μm in the reactions, TaVTC2 activity started to increase significantly when the concentration of TaVTC2 enzyme increased and exceeded 0.06 μm (Figure 4c). The above results revealed a nearly one to one stoichiometric ratio of TaVTC binding to NIb in vitro. Next, to further identify whether NIb influences TaVTC2 to bind its substrate GDP‐L‐galactose, microscale thermophoresis (MST) experiments were performed. A dissociation constant (*K*_d_) of 0.0027 mm was measured for the binding form TaVTC2+GDP‐L‐Gal, while the *K*_d_ value for the binding form was almost doubled to 0.0056 mm when TaVTC2 was pre‐incubated with Nib for 1 h. The results above demonstrated clearly that Nib in the reactions increased the *K*_d_ value of the binding form TaVTC2+GDP‐L‐Gal, and consequently, it reduced the affinity of VTC2 enzyme to its substrate GDP‐L‐Gal (Figure 4d). Additionally, isothermal titration calorimetry (ITC)‐binding experiments were further performed to verify this binding inhibition of NIb. The affinity between VTC2‐NIb and GDP‐L‐Gal discs was also decreased in comparison to the affinity between VTC2 and GDP‐L‐Gal (Figure 4e,f). The results showed that TaVTC2 enzyme activity was suppressed by NIb. Furthermore, we measured the concentration of AsA in WYMV‐infected YM158 plants, *TaVTC2*‐OE plants, and *TaVTC2*‐RNAi plants, with the resistant ‘Fielder’ and the susceptible YM158 as control. The AsA concentrations in both WYMV‐infected wheat plants and *TaVTC2*‐RNAi plants were significantly lower than in their respective controls (Figure 4h,i). Whereas the AsA concentration in *TaVTC2*‐OE plants was higher than the WT control (Figure 4g).

### TaVTC2 scavenges ROS accumulation promoting viral infection *in planta*

**Figure 5:**
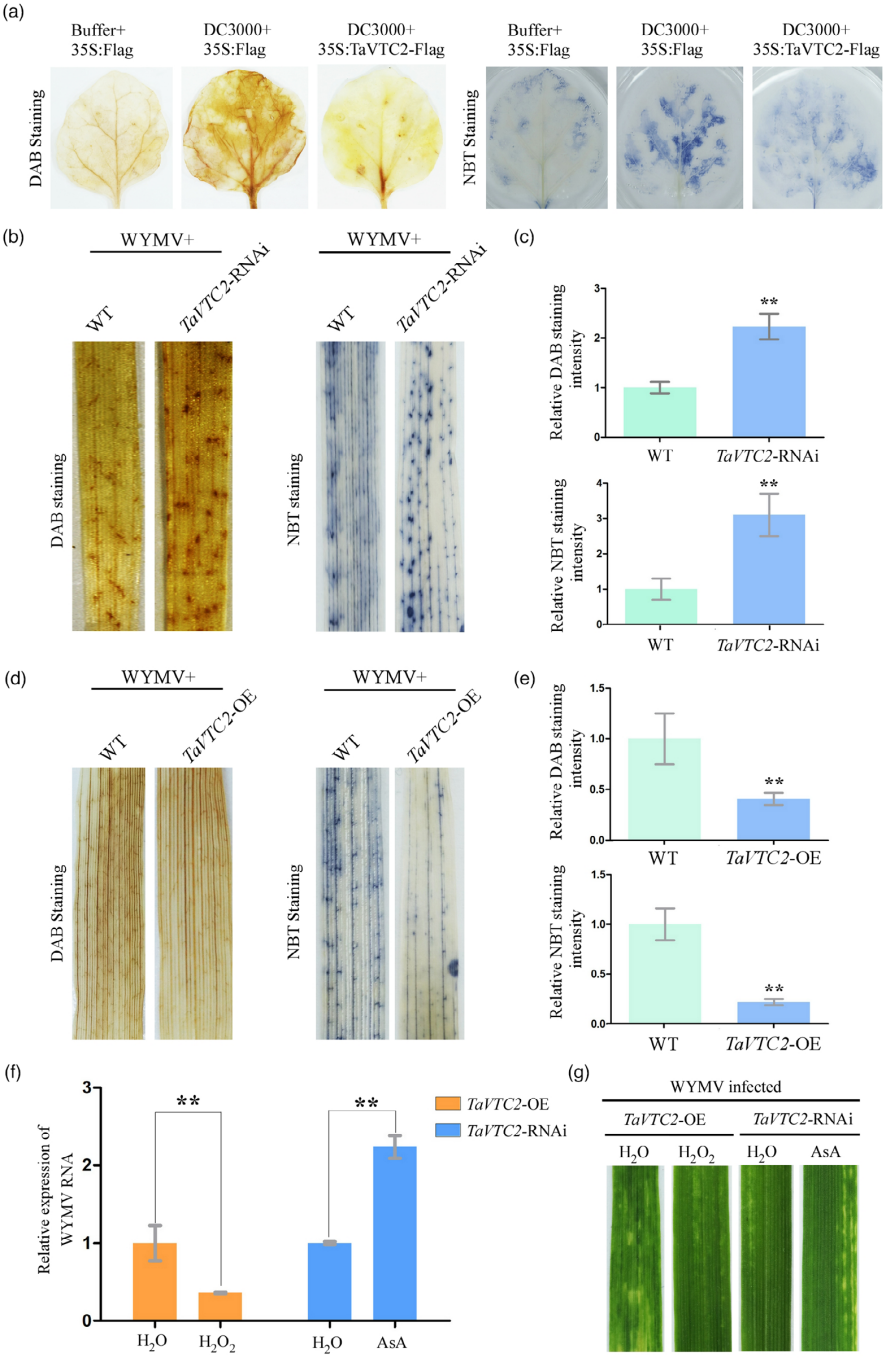
TaVTC2 scavenges ROS promoting viral infection *in planta*. (a) In situ detection of ROS in *N. benthamiana* leaves inoculated with Buffer + 35S:Flag, *P.s.t*. DC3000 + 35S:Flag and *P.s.t*. DC3000 + 35S:TaVTC2‐Flag, Buffer + 35S:Flag and *P.s.t*. DC3000 + 35S:Flag were used as negative and positive control respectively. (b, d) In situ detection of ROS in WYMV‐infected systemic leaves of WT (YM158)/*TaVTC2*‐RNAi, WT (Fielder)/*TaVTC2*‐OE at 14 days post‐inoculation (dpi). DAB and NBT staining were applied to analyse ROS production. (c, e) Histograms represent the relative DAB and NBT staining intensity as shown in (b, d) respectively. Values were obtained from measuring three representative images chosen from three biological replicates. Relative DAB and NBT staining intensity of WT leaves were set to 1. Statistical analysis was performed using Student's *t*‐test. *P < 0.05; **P < 0.01. (f) WYMV accumulation in the H_2_O or H_2_O_2_‐pretreated *TaVTC2*‐OE plants and H_2_O or AsA‐pretreated *TaVTC2*‐RNAi plants. Statistical analysis was performed using Student's *t*‐test. *P < 0.05; **P < 0.01; ns: no significant difference. (g) Phenotypes in the fourth leaves of H_2_O or H_2_O_2_‐pretreated *TaVTC2*‐OE plants and H_2_O or AsA‐pretreated *TaVTC2*‐RNAi plants inoculated with WYMV. Photographs were taken at 14 days post‐inoculation (dpi) with virus.

It was reported that AsA could detoxify ROS in plants (Conklin and Barth, 2004; Linster and Clarke, 2008). To explore the relationship between TaVTC2 and ROS, *N. benthamiana* leaves were examined to verify the association between TaVTC2 and ROS production. *Agrobacterium* (OD_600_ = 0.9) harbouring a 35S:TaVTC2‐Flag and an empty vector 35S:00 were infiltrated into leaves of *N. benthamiana* plants. *P. syringae* DC3000 was then infiltrated into these assayed leaves at 2 dpi with 35S:TaVTC2‐Flag or 35S:00. Plants infiltrated only with 35S:00 were used as negative controls and plants infiltrated with both 35S:00 and *P. syringae* DC3000 as positive controls. ROS accumulation in the leaves overexpressed with *TaVTC2* (Figure S4) was obviously reduced in comparison to the negative control after 1 dpi with *P. syringae* DC3000 (Figure 5a), indicating again that overexpressed *TaVTC2* can reduce ROS accumulation in tobacco leaves. Next, ROS accumulation in *TaVTC2*‐OE or *TaVTC2*‐RNAi plants after WYMV infection were analysed using DAB staining and NBT staining with WT plants as a control. After 7 dpi with WYMV, ROS accumulation in the leaves of *TaVTC2*‐RNAi wheat plants was significantly higher than in WT leaves (Figure 5b,c). Consistent with our expectation, ROS accumulation in the leaves of *TaVTC2*‐OE wheat plants was much lower than in WT leaves after 7dpi inoculated with WYMV (Figure 5d,e). To further illustrate possible roles of ROS and AsA in response to WYMV infection, exogenous H_2_O_2_ and AsA were used to treat *TaVTC2*‐OE or *TaVTC2*‐RNAi plants. Considering high concentration H_2_O_2_ may be led to mosaic symptoms in plant leaves (Wu *et al*., 2020), different concentration gradients of H_2_O_2_ (0.1 mm, 0.5 mm, 1 mm, 5 mm, and 10 mm) were applied to *TaVTC2*‐OE plants respectively. After 14 days, there were some mosaic symptoms on 5 mm and 10 mm H_2_O_2_‐treated wheat leaves while no mosaic symptoms on 0.1 mm, 0.5 mm, and 1 mm H_2_O_2_‐treated wheat leaves (Figure S5a). Therefore, 1 mm H_2_O_2_ was selected for exogenous spraying experiment. Twelve hours before WYMV inoculation, 1 mm AsA and 1 mm H_2_O_2_ were prepared and sprayed onto 10 *TaVTC2*‐RNAi or *TaVTC2*‐OE plants respectively. Before WYMV inoculation, five of the 10 pretreated plants were randomly selected for DAB staining. The result showed that the ROS accumulation in H_2_O_2_‐pretreated *TaVTC2*‐OE leaves was obviously higher than H_2_O‐pretreated leaves while the ROS accumulation in AsA‐pretreated leaves was lower than H_2_O‐pretreated *TaVTC2*‐RNAi leaves (Figure S5b). Then, the remaining five plants of *TaVTC2*‐RNAi or *TaVTC2*‐OE plants were used for WYMV inoculation respectively. After 14 dpi, *TaVTC2*‐OE wheat plants pretreated with exogenous H_2_O_2_ had less WYMV RNA accumulation and displayed weaker mosaic symptoms. However, *TaVTC2*‐RNAi plants pretreated with exogenously applied AsA had higher WYMV RNA accumulation and exhibited severe mosaic symptoms when compared with the H_2_O pretreated TaVTC2‐RNAi plants (Figure 5f,g). Thus, our results demonstrated that *TaVTC2* promoting WYMV infection may be through reducing the accumulation of ROS, which scavenged by AsA arisen from TaVTC2 activity in wheat.

### NIb interferes with the function of TaVTC2 in ROS scavenging in a dosage‐dependent manner

**Figure 6:**
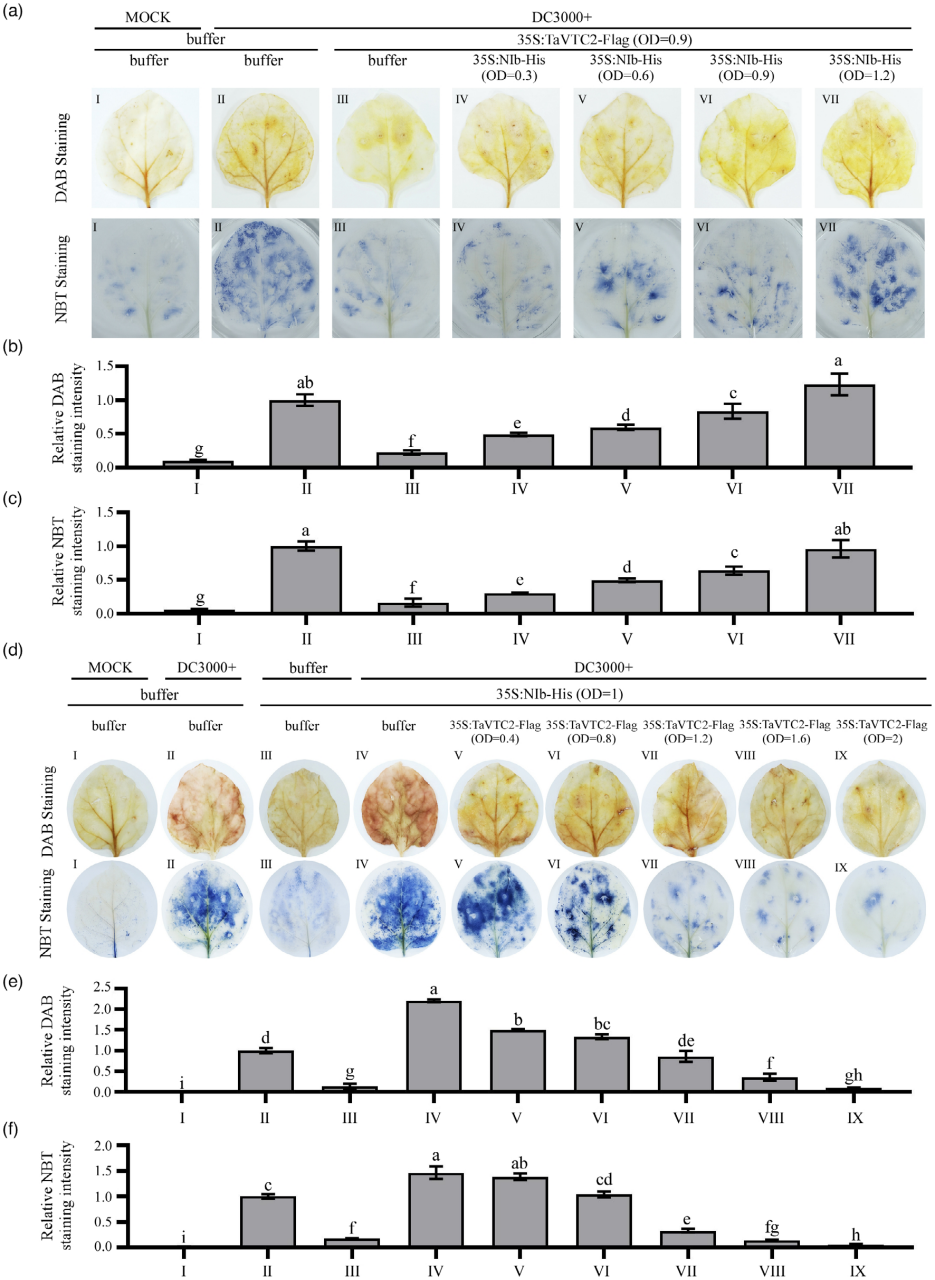
NIb interferes with the function of TaVTC2 in ROS scavenging in a dosage‐dependent manner. (a) In situ detection of ROS in a series of different pretreated *N. benthamiana* leaves (I–VII) which represent MOCK, *P.s.t*. DC3000, *P.s.t*. DC3000 + 35S:TaVTC2‐Flag (OD600 = 0.9), *P.s.t*. DC3000 + 35S:TaVTC2‐Flag (OD600 = 0.9) + 35S:NIb‐His (OD600 = 0.3), *P.s.t*. DC3000 + 35S:TaVTC2‐Flag (OD600 = 0.9) + 35S:NIb‐His (OD600 = 0.6), *P.s.t*. DC3000 + 35S:TaVTC2‐Flag (OD600 = 0.9) + 35S:NIb‐His (OD600 = 0.9), *P.s.t*. DC3000 + 35S:TaVTC2‐Flag (OD600 = 0.9) + 35S:NIb‐His (OD600 = 1.2) respectively. (b, c) Histograms represent the relative DAB and NBT staining intensity as shown in (a) respectively. (d) In situ detection of ROS in a series of different pretreated *N. benthamiana* leaves (I‐IX) which represent MOCK, *P.s.t*. DC3000, 35S:NIb‐His, *P.s.t*. DC3000 + 35S:NIb‐His (OD600 = 1), *P.s.t*. DC3000 + 35S:TaVTC2‐Flag (OD600 = 0.4) + 35S:NIb‐His (OD600 = 1), *P.s.t*. DC3000 + 35S:TaVTC2‐Flag (OD600 = 0.8) + 35S:NIb‐His (OD600 = 1), *P.s.t*. DC3000 + 35S:TaVTC2‐Flag (OD600 = 1.2) + 35S:NIb‐His (OD600 = 1), *P.s.t*. DC3000 + 35S:TaVTC2‐Flag (OD600 = 1.6) + 35S:NIb‐His (OD600 = 1), 35S:TaVTC2‐Flag (OD600 = 2) + 35S:NIb‐His (OD600 = 1) respectively. (e, f) Histograms represent the relative DAB and NBT staining intensity shown in (d). Values were obtained from measuring three representative images chosen from three biological replicates. Relative DAB and NBT staining intensity of *P.s.t*. DC3000 inoculated leaves were set to 1. Statistical analysis performed using Student's *t*‐test. *P < 0.05; **P < 0.01.

To explore whether the influence of NIb on TaVTC2 has an effect on ROS accumulation, C‐terminal His‐tagged NIb (35S:NIb‐His) and C‐terminal Flag‐tagged TaVTC2 (35S:TaVTC2‐Flag) recombinant plasmids were constructed and expressed alone or co‐expressed in *N. benthamiana* leaves following *agro‐infiltration*. We set up seven (I–VII) different experimental groups to quantify and analyse ROS production in wheat plants. Group I (mock‐inoculated) and II (only DC3000 inoculated) plants were, respectively, taken as the negative and positive control. Subsequently, the results of DAB and NBT staining showed that ROS accumulation was recovered and increased with increasing concentration of NIb in 35S::TaVTC2‐Flag‐overexpressed leaves (Figure 6a–c). The expression of WYMV NIb and TaVTC2 in these assayed *N. benthamiana* leaves was confirmed by Western blots (Figure S6a,b). To explore whether excessive TaVTC2 can also eliminate ROS while keeping a constant NIb, we set up nine (I–IX) different experimental groups to detect ROS production. The results of DAB and NBT staining revealed that excessive TaVTC2 could eliminate ROS in plants (Figure 6d–f). We also measured the protein expression of WYMV NIb and TaVTC2 in these assayed *N. benthamiana* leaves by Western blots (Figure S6c,d). Overall, these results indicate that WYMV NIb protein can inhibit the activity of TaVTC2 in vivo, leading to an increase in ROS accumulation in a dosage‐dependent manner.

### The accumulation of ROS in plants inhibits the WYMV infection

**Figure 7:**
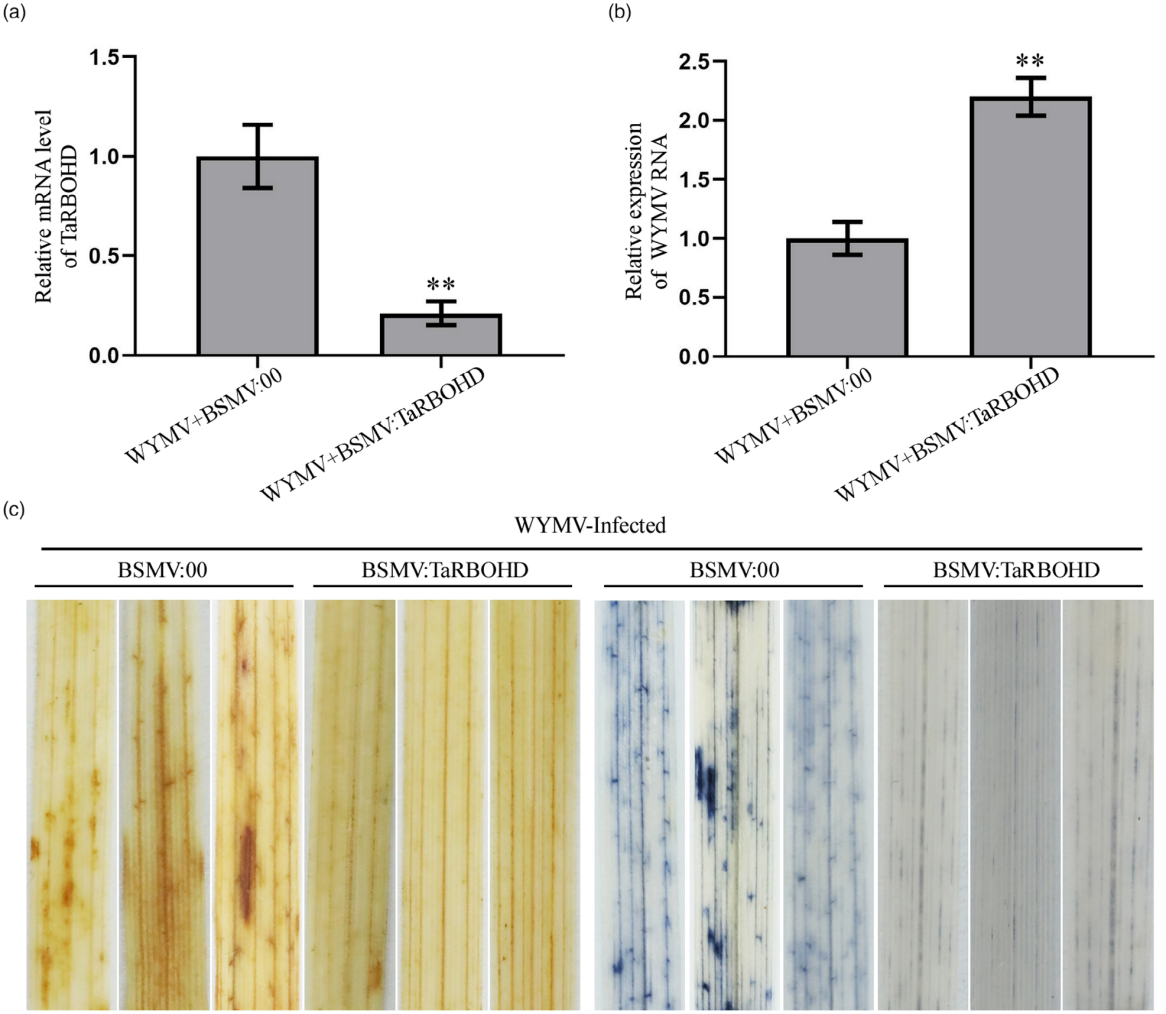
The accumulation of ROS in plants inhibits the WYMV infection. (a, b) Relative expression levels of TaRBOHD and WYMV CP in wheat plants co‐inoculated with WYMV + BSMV:00 and WYMV + BSMV:TaRBOHD. Each relative expression level is presented as mean ± SD from three biological triplicates. Statistical analysis performed using Student's *t*‐test. * *P* < 0.05; **P < 0.01; ns: no significant difference. (c) In situ detection of ROS in BSMV:00 and BSMV:TaRBOHD‐infected leaves at 14 d post‐inoculation (dpi). DAB and NBT staining were applied to analyse ROS production.

In order to determine whether ROS accumulation in wheat plants could affect WYMV infection, we first identified a homologous gene of *Arabidopsis respiratory burst oxidase homologue D* (i.e. *TaRBOHD*, TraesCS3A02G280200), which might regulate ROS production in wheat. Next, we constructed a recombinant plasmid, BSMV:TaRBOHD, and inoculated wheat seedlings with a series of RNA transcripts representing BSMV:TaRBOHD+WYMV or BSMV:00+WYMV. After 14 dpi, we detected the silencing level of the *TaRBOHD* gene using specific primers (Figure 7a) and measured the WYMV CP accumulation as well. The results showed that the mRNA accumulation of WYMV CP in TaRBOHD‐silenced wheat plants were significantly increased than in the control BSMV:00 wheat plants (Figure 7b). Furthermore, the DAB and NBT staining showed that almost no ROS was accumulated in TaRBOHD‐silenced wheat leaves, whereas typical ROS accumulated in BSMV:00 wheat leaves (Figure 7c). In summary, these results demonstrated that ROS accumulation in wheat plants can inhibit WYMV infection.

### Silencing of TaVTC2 confers broad‐spectrum disease resistance in wheat

**Figure 8:**
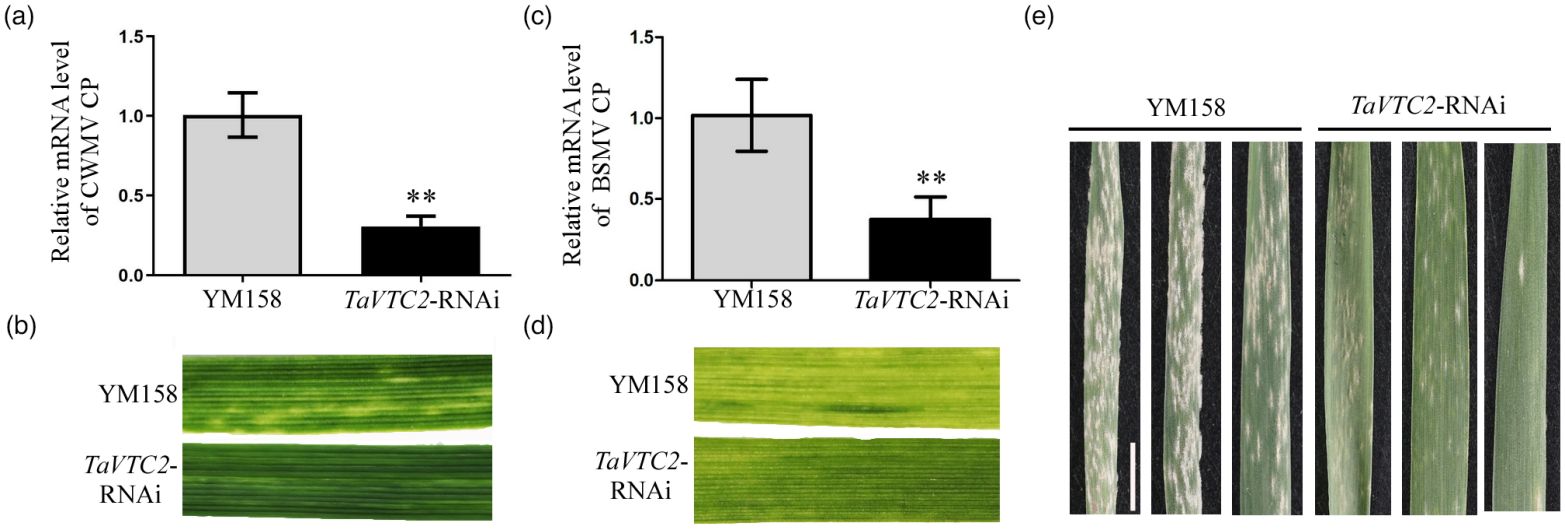
Silencing of TaVTC2 confers broad‐spectrum disease resistance in wheat. (a, c) CWMV (a) and BSMV (c) accumulation in YM158 and *TaVTC2*‐RNAi plants were determined by qRT‐PCR using CWMV CP‐specific primers and BSMV CP‐specific primers respectively. Each result (mean ± SD) was from three biological triplicates. Statistical analysis was performed using Student's *t*‐test. *P < 0.05; **P < 0.01. (b, d) Phenotypes in the fourth leaves of *TaVTC2*‐RNAi plants inoculated with CWMV or BSMV respectively. (e) Two‐week‐old YM158 and *TaVTC2*‐RNAi plants were inoculated with *Bgt* E09. Representative leaves were removed and photographed at 10 days post‐inoculation (dpi). Bar, 2 cm.

To further explore whether TaVTC2 mediates the host immunity formation, leading to broad‐spectrum disease resistance, two important RNA viruses, chinese wheat mosaic virus (CWMV) and BSMV of wheat were inoculated into *TaVTC2*‐RNAi plants. The accumulation of CWMV CP and BSMV CP was significantly reduced in *TaVTC2*‐RNAi plants (Figure 8a,c) and much less mosaic symptoms in leaves of *TaVTC2*‐RNAi plants compared to YM158 plants (Figure 8b,d). We also inoculated *TaVTC2*‐RNAi plants at the seedling stage with *Blumeria graminis f. sp. tritici* (*Bgt*) E09, which is one of the most devastating fungal diseases of wheat crops. *TaVTC2*‐RNAi plants displayed high resistance to *Bgt* E09 compared with YM158 plants, with only a few visible conidia produced when evaluated at 10 dpi (Figure 8e). In conclusion, this data suggest that *TaVTC2* knock‐down can activate host broad‐spectrum disease resistance.

## Discussion

Multiple physiological and biochemical processes have evolved in plants to respond and cope with various biotic stresses (Garcia‐Ruiz, 2019). Production of reactive oxygen species (ROS) like H_2_O_2_ is well‐known process for triggering ROS‐mediated plant immune responses (Li *et al*., 2017b; Mittler, 2017; Wu *et al*., 2017). For example, *Pseudomonas syringae* infection can increase ROS production through the activation of RBOHD in plants, while rice stripe virus (RSV) infection stimulates ROS production via increasing ascorbate oxidase activity, thus leading to a higher level of ROS accumulation and antiviral defence in both cases (Wu *et al*., 2017; Zhang *et al*., 2018). Abundant evidence shows that ROS accumulation in plants is determined by the AsA content, which is regulated by VTC2 (Linster and Clarke, 2008) (Luo *et al*., 2021); (Vidal‐Meireles *et al*., 2017). In this study, a VTC2 orthologue named as *TaVTC2* gene was cloned from wheat and sequenced, showing a close identity with homologue genes from other plant species (Figure 1a). The implication of this gene in viral infection was clearly demonstrated by our results that overexpression of *TaVTC2* enhanced the WYMV infection in the transgenic wheat plants, whereas the *TaVTC2* knocked down wheat plants exhibited less mosaic symptoms, so less infection or more resistance to WYMV (Figure 2). Indeed, AsA concentration was increased in the *TaVTC2*‐OE wheat plants, while decreased in the *TaVTC2*‐RNAi and WYMV‐infected wheat plants (Figure 4g–i). A previous study reported that AsA content in an *A. thaliana vtc2* mutant decreased by 70%–80% but its disease resistance to pathogens was significantly enhanced (Conklin and Barth, 2004). Therefore, our results were consistent with the case in Arabidopsis *vtc2* mutant, demonstrating that *TaVTC2* can positively regulate the WYMV infection in wheat via regulating AsA synthesis.

The conclusion above was further supported by our experiment on tobacco plants. Overexpression of *TaVTC2* in tobacco leaves can scavenge the *P.s.t*. DC3000‐mediated ROS burst (Figure 5a), indicating that ROS was quenched by AsA arisen from this TaVTC2 overexpression. The accumulation of ROS in plants is usually due to the infection of pathogens (Smirnoff and Arnaud, 2019). We found that WYMV infection indeed caused the ROS accumulation in wheat leaves (Figure 5b,d), which is consistent with the result in our recent study of the inhibition of ROS scavenging pathway with WYMV, deriving small interfering RNA which resulted in ROS accumulation (Liu *et al*., 2021). However, the ROS level did not increase in *TaVTC2*‐OE leaves after WYMV infection, while this was not the case in the WT leaves. Consistently, the *TaVTC2*‐RNAi leaves displayed strikingly higher ROS level than WT leaves (Figure 5b–e). Furthermore, exogenously applied AsA restored the WYMV infection and mosaic symptoms in *TaVTC2*‐RNAi plants (Figure 5f,g), and silencing *TaRBOHD* in wheat plants (ROS production is suppressed) promoted WYMV infection (Figure 7). Meanwhile, the expression of *TaRBOHD* in *TaVTC2*‐OE and *TaVTC2*‐RNAi lines upon WYMV infection had no significant change (Figure S7), which suggests that the TaVTC2‐mediated changes in ROS may have no signal feedback or independent to TaRBOHD‐mediated ROS production. This data further proved that TaVTC2 can positively regulate the viral infection through AsA‐mediated antioxidant pathway.

ROS are usually considered to be a positive regulator of plant antiviral defences (Deng *et al*., 2016). Citrus tristeza virus p33 protein is hijacked by citrus miraculin‐like protein then induces cellular oxidative stress in defence against viral infection (Sun *et al*., 2021). Tobacco mosaic virus p50 could be recognized by the tobacco N protein then triggers ROS burst, accompanied by a hypersensitive response (Caplan *et al*., 2008; Whitham, 1995). However, some evidence also indicate that viruses can suppress antioxidant systems to increase ROS accumulation then promote viral infection. BSMV γb protein subverts NTRC‐mediated chloroplast antioxidant defences to create an oxidative microenvironment for viral replication (Wang *et al*., 2021). RCNMV replication also requires RBOHB‐mediated ROS accumulation (Hyodo *et al*., 2017). These may be due to the fact that suitable oxidized microenvironments are favourable for some viruses replication like flavivirus and alphavirus (Gullberg *et al*., 2015). In this study, silencing *TaRBOHD* (ROS production is suppressed) or exogenously applied AsA both promoted WYMV infection, while exogenously applied H_2_O_2_ inhibited WYMV infection (Figures 5 and 7). These results indicated that the accumulation of ROS in mesophyll cells may activate the host antiviral defence response then defence against WYMV infection in wheat.

What's more, ROS offers wide possibilities for broad‐spectrum disease resistance in plants (Hu *et al*., 2021; Li *et al*., 2019). In this study, we found that the *TaVTC2*‐RNAi transgenic wheat lines were resistant to CWMV, BSMV, and *Bgt* due to their high ROS production upon infection (Figure 8). However, a higher level of crop resistance often led to yield penalties. Therefore, it is particularly important to improve disease resistance of crops while maintaining their good agronomic traits, which is one of the main challenges for crop disease resistance breeding. Interestingly, a natural mutation of a rice transcription factor confers broad‐spectrum resistance to rice blast with little impact on rice growth and yield penalty (Li *et al*., 2017a). Our observations of the agronomic performance of *TaVTC2*‐RNAi lines in the field revealed that the seed number per ear and 1000 kernel weight of *TaVTC2*‐RNAi lines were slightly decreased (Figure S8a–c). This may be caused by the decrease in AsA content in transgenic plants, which leads to a decrease in its detoxification effect during development. Unfortunately, we failed to generate a TaVTC2‐knockout mutant and could not examine the critical role of TaVTC2 in the growth and development of wheat plants. Thus, we believe that these *TaVTC2*‐RNAi lines could still serve as potential quality germplasm resource for future breeding projects.

It has been reported that in addition to the roles as an RNA polymerase of the VRC and a component recruiter, NIb may function as a suppressor of host defence response (Dufresne *et al*., 2008; Shen *et al*., 2020). TuMV NIb has been identified as a suppressor of host defence response that specifically counter SUMO3‐activated NPR1‐mediated immunity signalling pathway. Because of NIb's important role in viral infection, plants have also evolved new strategies to monitor NIb protein. In pepper, the dominant resistance gene *Pvr4* recognizes NIb of six potyviruses to trigger this resistance (Fellers *et al*., 2002; Janzac *et al*., 2009; Kim *et al*., 2018). In our study, we found TaVTC2 can recognize WYMV NIb protein, then activate ROS‐mediated antiviral immunity. Meanwhile, our previous study showed that WYMV NIb interacts with wheat light‐induced protein (TaLIP) to facilitate viral infection through interfering the ABA signalling pathway (Zhang *et al*., 2019). Therefore, we detected the mRNA expression level of a series of ABA signalling pathway genes in *TaVTC2‐OE* and *TaVTC2‐RNAi* lines upon WYMV infection. And the expression level of these genes has no significant changes in these lines (Figure S9), which suggests that NIb‐TaVTC2‐mediated defence pathway is independent to that of NIb‐TaLIP. Previous study has showed that NIb can be recognized by the autophagy protein Beclin 1 and degraded through autophagosomes inhibiting viral infection (Li *et al*., 2018a). On the contrary, host could encode RPL1 to compete with Beclin1 to bind NIb, reduces Beclin1‐mediated NIb degradation, and enhances viral infection (Cheng *et al*., 2021). It is the co‐evolutionary arms race that leads to an offensive and defensive balance between plants and viruses. Taken together, this study reveals a novel potential mechanism for host targeting the NIb protein to control viral infection.

**Figure 9:**
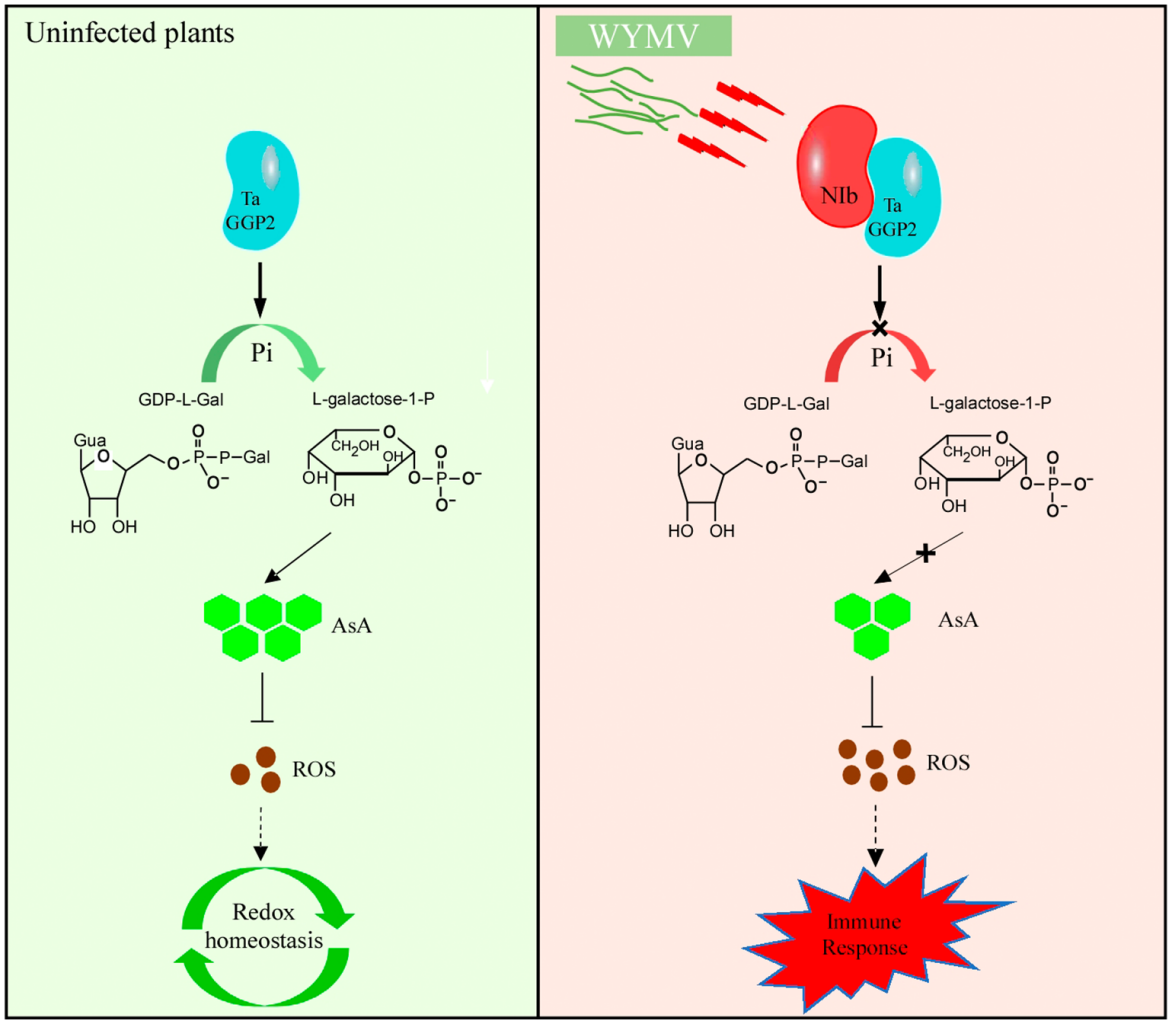
Model illustrating the novel role of TaVTC2 in regulating plant antioxidant defence through recognizing the NIb. Expression of TaVTC2 in healthy wheat plants ensures the synthesis of AsA and maintains redox homeostasis in plant cells (left panel). In WYMV‐infected leaves, TaVTC2 protein recognizes and binds to WYMV NIb protein. This interaction down‐regulates TaVTC2 catalytic activity, leading to the reduction of L‐galactose‐1‐P and the decrease of ASA production. Consequently, more ROS accumulated in plant cell results in a broad‐spectrum resistance to limit pathogen invasion.

Here, we propose a model illustrating a novel role of TaVTC2 in regulating plant antioxidant defence through recognizing the NIb as an elicitor to induce innate immunity in wheat plants (Figure 9). A successful infection by a plant virus results from the complex molecular interplay between the host plant and the invading virus. Here, we found TaVTC2 can recognize NIb to inhibit WYMV infection, but it is unknown whether NIb could evade the recognition of TaVTC2 through some strategies which remains to be further studied. Moreover, ROS signalling is highly integrated with hormonal signalling networks (Mittler *et al*., 2011). TaVTC2‐mediated ROS signal whether affects hormonal signalling pathways or not needs to be further explored. In summary, our finding has expanded the current understanding of the functional roles of *potyvirus* NIb in the formation of innate immunity in plants, and the functional identification of TaVTC2‐NIb interaction assist in the development of novel effective antiviral strategies for sustainable crop production.

## Methods

### Plant materials and growth conditions

A WYMV‐susceptible wheat (*Triticum aestivum*) cultivar, cv. Yangmai 158 (YM158), and two WYMV‐resistant cultivars, cv. Ningmai 9 (NM9) and cv. *Fielder*, were used in our experiments. All wheat seedlings were grown in a glasshouse at room temperature. WYMV‐infected wheat seedlings were grown in a climate chamber at 10 ± 2 °C with a 16 h light/8 h dark photoperiod. For field tests, YM158, NM9, ‘*fielder’*, and three transgenic wheat lines were grown in a field nursery that has a WYMV infection history in Junan, Shandong Province, China, from 2019 to 2021.

### Wheat transformation

The designed fragment of TaVTC2 CDS were cloned individually and inserted into the *BamH* I and *Sal* I sites followed by the pCAMV35S promoter in the expression vector pCAMV35S:00 to produce pCAMV35S:TaVTC2. For TaVTC2‐RNAi transgenic wheat plants, the intron of the maize alcohol dehydrogenase 1 (adh1) gene flanked by the 300 bp fragment of TaVTC2 in sense and antisense orientations was used to construct a hairpin RNA cassette (TaVTC2‐pAHC25) using the restriction sites of *SmaI* and *SacI* under control of the Ubi promoter and terminated by the NOS terminator. The vector pAHC20 containing a selective marker gene (herbicide resistance gene) was co‐transformed with pCAMV35S:TaVTC2 or TaVTC2‐pAHC25 into immature wheat embryos by particle bombardment. Selection and generation of transgenic wheat plants were carried out as previously described (Chen *et al*., 2014).

### RNA isolation, TaVTC2 cloning, and qRT‐PCR analysis

Leaf samples were frozen in liquid nitrogen immediately after collection and stored at −80 °C until use. Total RNAs were extracted from plants using FastPure Universal Plant Total RNA Isolation Kit (Vazyme Biotech Co., Ltd) and stored at −80 °C. The integrity and concentration of each total RNA sample was detected using Mops gel electrophoresis and a NanoDrop 1000 spectrophotometer (Thermo Fisher Scientific, Wilmington, DE). Plant cDNA was synthesized through total RNA reverse transcription using a First Strand cDNA Synthesis Kit (Toyobo, Kita‐ku, Osaka, Japan). For length of TaVTC2, CDS was cloned through RT‐PCR with paired primers list in Table S1. Quantitative real‐time (qRT) PCR analysis was performed using an ABI Q5 Sequence Detection System (Applied Biosystems, Foster City, CA) with an AceQ qPCR SYBR Green Master Mix (Vazyme, Nanjing, Jiangsu, China). At least three biological triplicates were used for each assay. The *T. aestivum* cell division cycle (CDC) gene (Accession Number: XM_020313450) and the *N. benthamiana* actin gene were used as internal reference genes for analysis to calculate the fold changes in gene expression. The fold changes were calculated using the 2^−ΔΔCT^ method (Schmittgen and Livak, 2008). All gene‐specific primers for qRT‐PCR are shown in Table S1.

### Co‐immunoprecipitation assays and Western blot

Co‐immunoprecipitation (Co‐IP) assays were performed as described previously (Zhang *et al*., 2019). About 0.5 g of agroinfiltrated leaf tissue was frozen in liquid nitrogen, ground to a fine powder, and then thawed in a plant protein extraction buffer (100 mM Tris–HCl, pH 8.8, 60% SDS, and 2% B‐mercaptoethanol) with protease inhibitor cocktail tablets (one tablet per 50 mL buffer). The mixture was centrifuged at 18 000 ***g*** for 10 min at 4 °C. Each supernatant (500 μL) was mixed with 45 μL of anti‐GFP‐conjugated agarose beads (Sigma) and incubated at 4 °C for 1.5 h with gentle shaking. Agarose beads were pelleted and washed three times with the Co‐IP buffer. The resulting pellets were boiled in SDS loading buffer. For the immunoblot, proteins were separated on 10% SDS‐PAGE gels (SurePAGE™, Genscript, M00652) through electrophoresis, and then transferred to NC membranes using eBlot™ L1 (Genscript, L00686C). The blots were probed with an anti‐HA (1 : 5000) and an anti‐GFP (1 : 5000), followed by an HRP‐conjugated secondary antibody (HUABIO, HA1006).

### GST pull‐down assays

Purified TaVTC2‐GST protein was incubated with purified His‐tagged NIb protein at room temperature for 30 min. Next, 25 μL GST‐Trap agarose (ChromoTek) was added into reaction system (50 mm Tris–HCl, pH 9.0, 300 mm NaCl, 1.5% glycerol, 0.6% Triton X‐100, and 0.1% Tween 20), and then incubated at 4 °C for 2 h. Afterwards, beads were collected and washed three times with Tris‐buffered saline solution (TBS; 10 mm Tris–HCl pH 8.0, 150 mm NaCl). The washed beads were boiled in 1× SDS loading buffer, and the proteins were run using SDS‐PAGE for Western blot with anti‐GST or anti‐His antibodies (TransGene).

### Droplet digital PCR

The total genomic DNA of transgenic wheat lines was isolated using the CTAB method as described previously (Chen *et al*., 2014). The *TaPINb* (PUROINDOLINE‐b) gene was used as the reference gene. The pCaMV35S promoter, which is often used to control gene expression in transgenic wheat plants, was detected using the specific probe and primers as shown in Table S1. The gene copies detection method was performed as described in the previous study (Collier *et al*., 2017).

### Yeast two‐hybrid assay

Yeast two‐hybrid (Y2H) assays were performed following the method described in the Takara protocol handbook. The full length of TaVTC2 was cloned and fused to pGADT7. WYMV NIb and four fragments of WYMV NIb were fused to the pGBKT7. Yeast cells (strain Y2H Gold) carrying the co‐transformed plasmids were plated onto a low‐stringency selective medium lacking tryptophan and leucine (SD/−Trp‐Leu) to confirm the transformation and then plated onto a high‐stringency selective medium lacking tryptophan, leucine, histidine, and adenine (SD/−Trp‐Leu‐His‐Ade) to analyse the interaction.

### BSMV‐based VIGS and mechanical friction inoculation of BSMV RNAs

BSMV‐based VIGS was performed as described previously (Yang *et al*., 2020a). In brief, BSMV RNA α, β, and γ were linearized to plasmid DNAs (pBSMV α, pBSMV β, and pBSMV γ) and transcribed in vitro, respectively, using the Ribo MAX™ Large Scale RNA Production Systems‐T7 and the Ribo m7G Cap Analog (both by Promega) following the manufacturer's instructions. The resulting BSMV α, β, and γ RNA transcripts were mixed in a ratio 1 : 1 : 1, then 3 μL mixed BSMV RNAs was diluted with 7 μL inoculation buffer (0.06 M potassium phosphate, 0.1 M glycine, 1% bentonite, 1% sodium pyrophosphate decahydrate, and 1% celite, pH 8.5), and rub‐inoculated to the leaf 2 (bottom up) of a wheat seedling at the three‐leaf stage. The inoculated seedlings were grown inside a dark growth chamber at 25 °C and high humidity for 24 h, and then under a 16 h : 8 h, light:dark photoperiod.

### Inoculation of wheat seedlings with WYMV RNAs and CWMV RNAs

WYMV RNA1 and RNA2 were linearized with *Spe*I restriction digestion and transcribed in vitro using the Promega Ribo MAX™ Large Scale RNA Production Systems‐Sp6 and the Ribo m7G Cap Analog following the manufacturer's instructions. After adding approximately 1 μg of linearized plasmid template, the reaction mixtures were incubated at 37 °C for 1 h. Transcripts WYMV RNA1 and RNA2 were mixed in a 1 : 1 ratio and diluted twofold with RNase‐free H_2_O. Next, 2 μL of mixed WYMV RNAs were diluted with 8 μL of inoculation buffer (0.06 M potassium phosphate, 0.1 M glycine, 1% bentonite, 1% sodium pyrophosphate decahydrate, 1% celite, and pH 8.5) and used to rub‐inoculate the second leaf (bottom up) of a wheat seedling at the three‐leaf stage. The inoculated seedlings were grown at 8 °C and 80% humidity in a constant temperature and humidity incubator.

### Wheat powdery mildew and infection experiments

*Blumeria graminis* f. sp. *tritici* (*Bgt*) isolate E09, originally collected in Beijing, inoculated to wheat seedings were raised in small pots filled with pine bark/loam‐based potting mix. Each experiment had three replications. Average temperatures for daytime and night in glasshouse were 25 ± 2 and 16 ± 2 °C respectively; no supplemental light throughout experiment. The responses of wheat seedings to *Bgt* E09 were determined at 15 days post‐inoculation.

### Histochemical staining of ROS

The histochemical staining of ROS was performed using 3, 3′‐diaminobenzidine (DAB). *N. benthamiana* leaves were stained with 1 mg/mL of DAB–HCl solution overnight. The morphological bottom of wheat leaves was inserted into DAB solution for histochemical staining by transpiration overnight. The relative intensity of DAB staining was calculated as described in previous studies (Hui *et al*., 2017; Zhang *et al*., 2018). IMAGEJ software (http://rsbweb.nih.gov/ij) was used to measure the relative intensity of DAB staining.

### GDP‐L‐galactose phosphorylase assays

The GDP‐L‐galactose used in the assays was synthesized by Qiyue Biological Co., Ltd, Xi'an, Shanxi Province, China, and L‐galactose 1‐phosphate was synthesized by Zhenzhun Biological Co., Ltd, Shanghai, China. GDP purchased from MCE. The phosphorylase activity assay of recombinant TaVTC2 protein was performed as described previously with slight modifications (Linster *et al*., 2007). In brief, TaVTC2 activity was evaluated by measuring GDP or L‐galactose 1‐phosphate production after incubation with 115 μm GDP‐L‐galactose in a reaction buffer (50 mm Tris–HCL pH = 7.5, 5 mm NaH_2_PO_4_, 2 mm MgCl_2_, 10 mm NaCl, and 1 mm DTT). Next, TaVTC2 enzyme or TaVTC2 enzyme and NIb were added to the reaction mixture. The reaction was maintained at 26 °C for 60 min and then stopped by boiling at 98 °C for 3 min. GDP produced in the reaction was detected by HPLC according to the method described previously (Linster *et al*., 2007).

### Microscale thermophoresis assay

Microscale thermophoresis (MST) assay method was described in a previous study (Wienken *et al*., 2010). Briefly, the affinity of the purified TaVTC2 for GDP‐L‐Gal was determined using Monolith NT.115 (NanoTemper Technologies). MST labelling of TaVTC2 was conducted in PBS solution containing a Monolith NT protein labelling kit RED according to the manufacturer's instructions (NanoTemper Technologies). Samples were then loaded into NanoTemper hydrophilic‐treated capillaries. The resulting samples were analysed by the manufacturer using NanoTemper analytical software to estimate their equilibrium dissociation constant *K*_d_ values.

### Isothermal titration calorimetry

The isothermal titration calorimetry (ITC) binding experiments were performed using the same method as the previous study (Li *et al*., 2018b) and used an ITC 200 Micro Calorimeter (GE Healthcare) at 20 °C. Briefly, the buffer contained 10 mm Tris–HCl and 150 mm sodium chloride (pH 7.5). The GDP (0.01 mm) w titrated into TaVTC2 (20 μm) in a 200 μL sample cell using a 40 μL microsyringe as follows: 0.4 μL for the first injection and 2 μL for the next 19 injections at intervals of 150 s. The integrated heat data were analysed using the one‐set‐of‐sites model in MicroCal Origin 7.0 according to the manufacturer's instructions. Data are presented as means ± SDs of triplicate assays.

### Enzyme kinetic analysis of recombinant TaVTC2

Recombinant TaVTC2 activity was measured in a coupled assay based on phosphate‐dependent GDP formation (Dowdle *et al*., 2010). The assay mixture (1.0 mL) contained 50 mm HEPES, pH 6.9, 50 mm KCl, 2.5 mm MgCl_2_, 1 unit pyruvate kinase, 0.1 μm TaVTC_2_, 0.25 mm NADH, 2 units lactate dehydrogenase, 2 mm phosphoenolpyruvate, 5 mm K_2_HPO_4_, and 0.01–0.6 mm GDP‐L‐gal. Phosphate‐dependent NADH oxidation was monitored at 339 nm at 20 °C.

### Measurement of AsA concentration

AsA concentration was measured as described in a previous study (Li *et al*., 2010). Briefly, wheat seedlings were homogenized in 6% trichloroacetic acid (TCA) and then centrifuged for 5 min at 13 000 ***g*** (4 °C). A total quantity of 0.2 mL sample (6% TCA was used as a blank) was added to a mixture with 0.2 M phosphate buffer (pH 7.4), 0.2 mL ddH_2_O, 1 mL 6% TCA, 0.8 mL 42% H_3_PO_4_, 0.8 mL 4% 2, 2′‐bipyridyl, and 0.4 mL 3% FeCl_3_. The assay tube was incubated at 42 °C for 1 h and the absorbance was read at 525 nm.

### AsA and H_2_O_2_ treatment

H_2_O_2_ and AsA were both purchased from Sigma‐Aldrich (http://www.sigmaaldrich.com). H_2_O_2_ and AsA were dissolved in dd H_2_O, prepared and dilute to different concentration gradients. Then, the prepared dilutions were sprayed onto wheat leaves for 12 h before WYMV inoculation. Wheat leaves sprayed by ddH_2_O were used as control treatment.

## Conflict of interest statement

The authors declare no conflict of interest.

## Author contributions

J. Y., J. C., and W. L. designed experiments. T. Z., H. H., Z. W., T. F., L. Y., J. Z., W. G., Y. Z., M. S., P. L., and K. Z. performed the experiments. All authors analysed and discussed the results. T. Z. and J. Y. wrote the article.

## Supporting information


**Figure S1** Identification of positive and single copy number wheat lines in an array of crops.
**Figure S2** Field assessment of T3 transgenic lines of *TaVTC2*‐OE for agronomic traits.
**Figure S3** Detection of virus infection efficiency and TaVTC2 silencing efficiency.
**Figure S4** Western blot analysis of the TaVTC2‐Flag.
**Figure S5** Exogenous application of H_2_O_2_ to wheat plants and DAB staining.
**Figure S6** Western blot analysis of TaVTC2 protein and NIb protein expression.
**Figure S7** Detection of *TaRBOHD* mRNA expression.
**Figure S8** Field assessment of transgenic lines of *TaVTC2*‐RNAi for agronomic traits.
**Figure S9** Relative mRNA expression level of ABA signalling pathway genes.

**Table S1** Primers used in this research.
