# Supplementary material for: Wheat yellow mosaic virus NIb targets TaVTC2 to elicit broad‐spectrum pathogen resistance in wheat
Source: Plant Biotechnol J. 2023 Feb 14;21(5):1073–88. doi: 10.1111/pbi.14019 (PMC10106851; doi:10.1111/pbi.14019)
Supplement: Supplementary file 1 — Figure S1 Identification of positive and single copy number wheat lines in an array of crops. Figure S2 Field assessment of T3 transgenic lines of TaVTC2‐OE for agronomic traits. Figure S3 Detection of virus infection efficiency and TaVTC2 silencing efficiency. Figure S4 Western blot analysis of the TaVTC2‐Flag. Figure S5 Exogenous application of H2O2 to wheat plants and DAB staining. Figure S6 Western blot analysis of TaVTC2 protein and NIb protein expression. Figure S7 Detection of TaRBOHD mRNA expression. Figure S8 Field assessment of transgenic lines of TaVTC2‐RNAi for agronomic traits. Figure S9 Relative mRNA expression level of ABA signalling pathway genes. [file PBI-21-1073-s002.docx]

**SUPPORTING INFORMATION:**


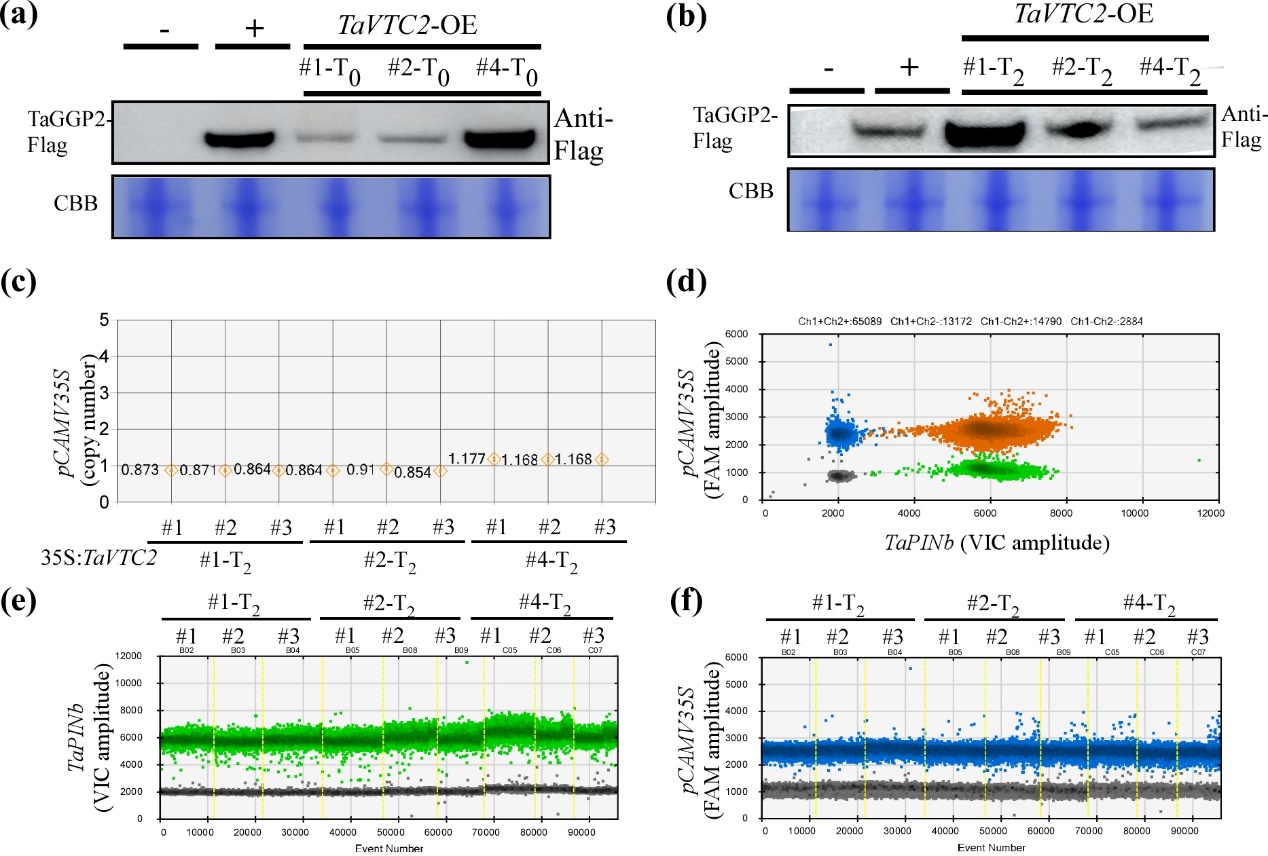


**Figure S1** **Identification of positive and single copy number wheat lines in an array of crops.** (a, b) Three positive wheat lines of *TaVTC2*-OE plants (#1, 2, 4) were confirmed by Western blot in T1 and T2 generations. negative control, healthy Fielder plants; positive control, Tobacco leaf transient expressed 35S:TaVTC2-Flag. Coomassie brilliant blue (CBB) large stained rubisco gel was used to show protein loadings. (c) Display of the calculated the transgene copy number values and error for Three positive wheat lines of *TaVTC2*-OE-T2 plants (#1, 2, 4) are shown. The wheat PINb VIC gene (PUROINDOLINE-b, TaPINb) was used as reference gene. The copy number and error values (Poisson 95% confidence interval) were calculated by the QuantaSoft™ software. (d) The droplets visualized in two-dimensions for *TaVTC2*-OE-T2 plants. (e, f) One-dimensional plot of droplets measurement. Negative droplets are shown in black.


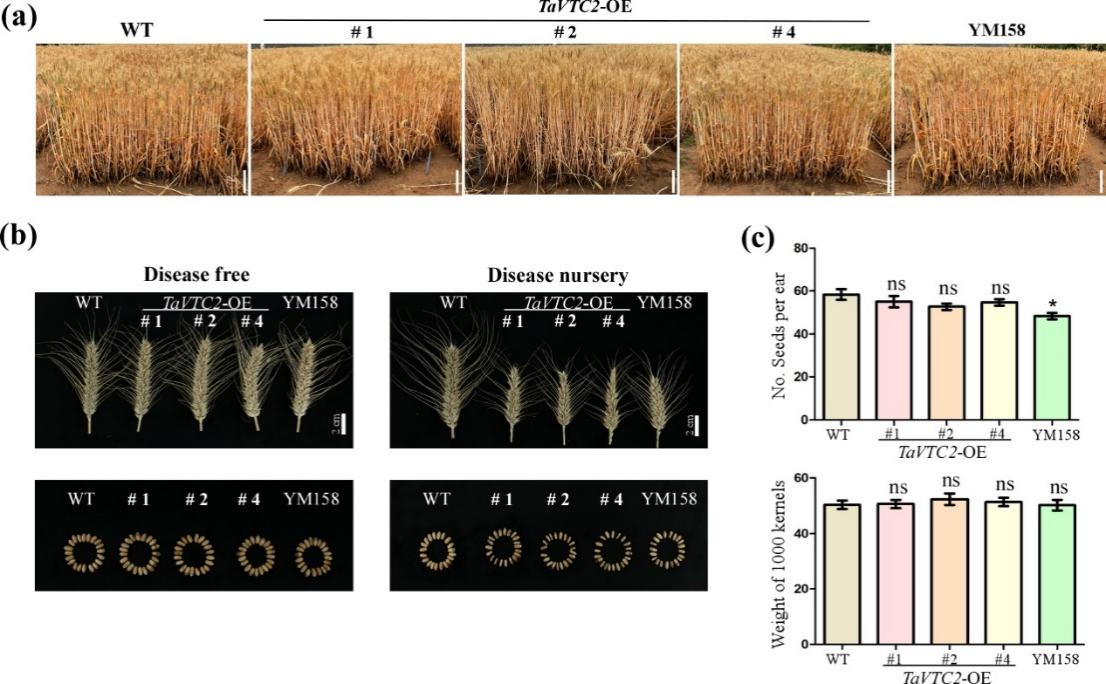


**Figure S2** **Field assessment of T3 transgenic lines of *TaVTC2*-OE for agronomic traits.** (a) Field assay results showing *TaVTC2*-OE, Fielder (WT), and YM158 plants at maturity. Bar, 30 cm. (b) Comparison of seed quality and wheat ears among transgenic plants, YM158, and Fielder (WT) in the disease nursery or a disease-free field. Bar, 2.0 cm. (c) Analyses of seed number per wheat ear and 1000-kernel weight of the *TaVTC2*-OE-T3, Fielder (WT), and YM158 plants at maturity.


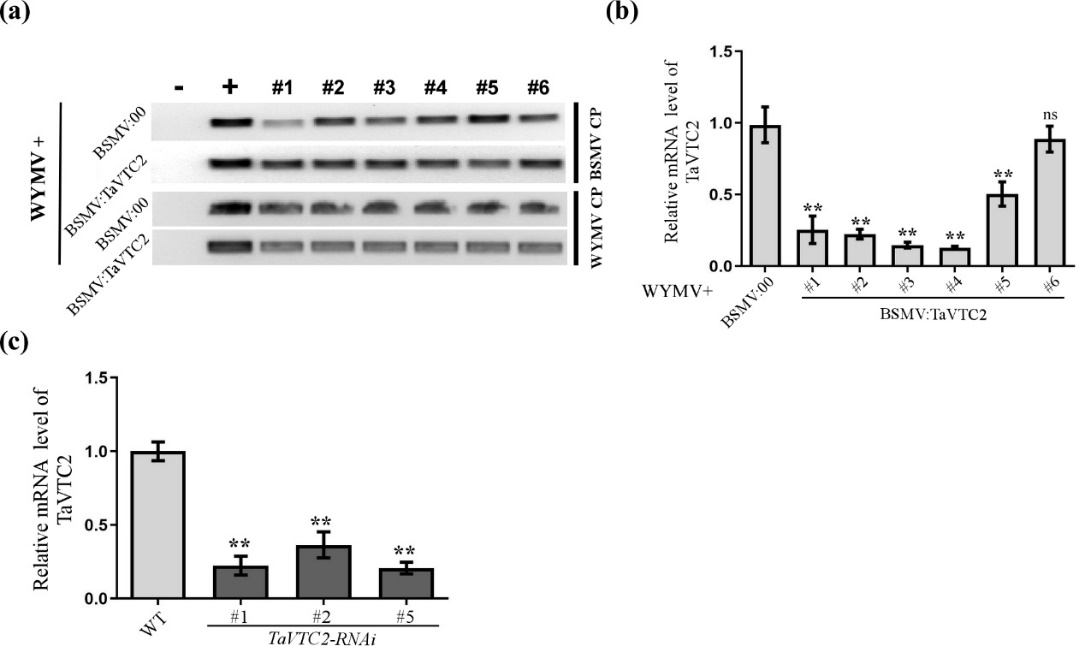


**Figure S3** **Detection of virus infection efficiency and TaVTC2 silencing efficiency.**

(a) Detection of BSMV and WYMV infection in twenty-four assayed plants by RT-PCR using BSMV and WYMV CP specific primers. (b, c) Relative expression level of TaVTC2 in six BSMV: TaVTC2 plants and *TaVTC2*-RNAi plants. Total RNA from BSMV:00 and WT (YM158) plants were used as control, respectively. Each relative expression level is presented as mean ± SD from three biological samples and each biological sample had three technical replicates. Statistical analyses were done using Student’s t-test. *, P < 0.05; **, P < 0.01; ns, no significant difference.


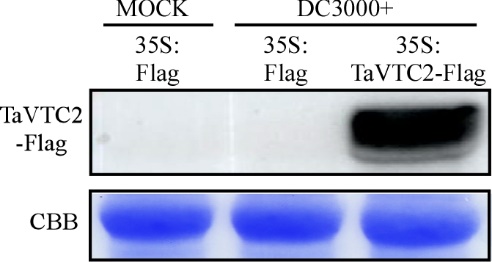


**Figure S4** **Western blot analysis of the TaVTC2-Flag.** Detection of TaVTC2-Flag in tobacco leaves of (Figure 5a) with anti-Flag antibody. Coomassie brilliant blue (CBB) large stained rubisco gel was used to show protein loadings.


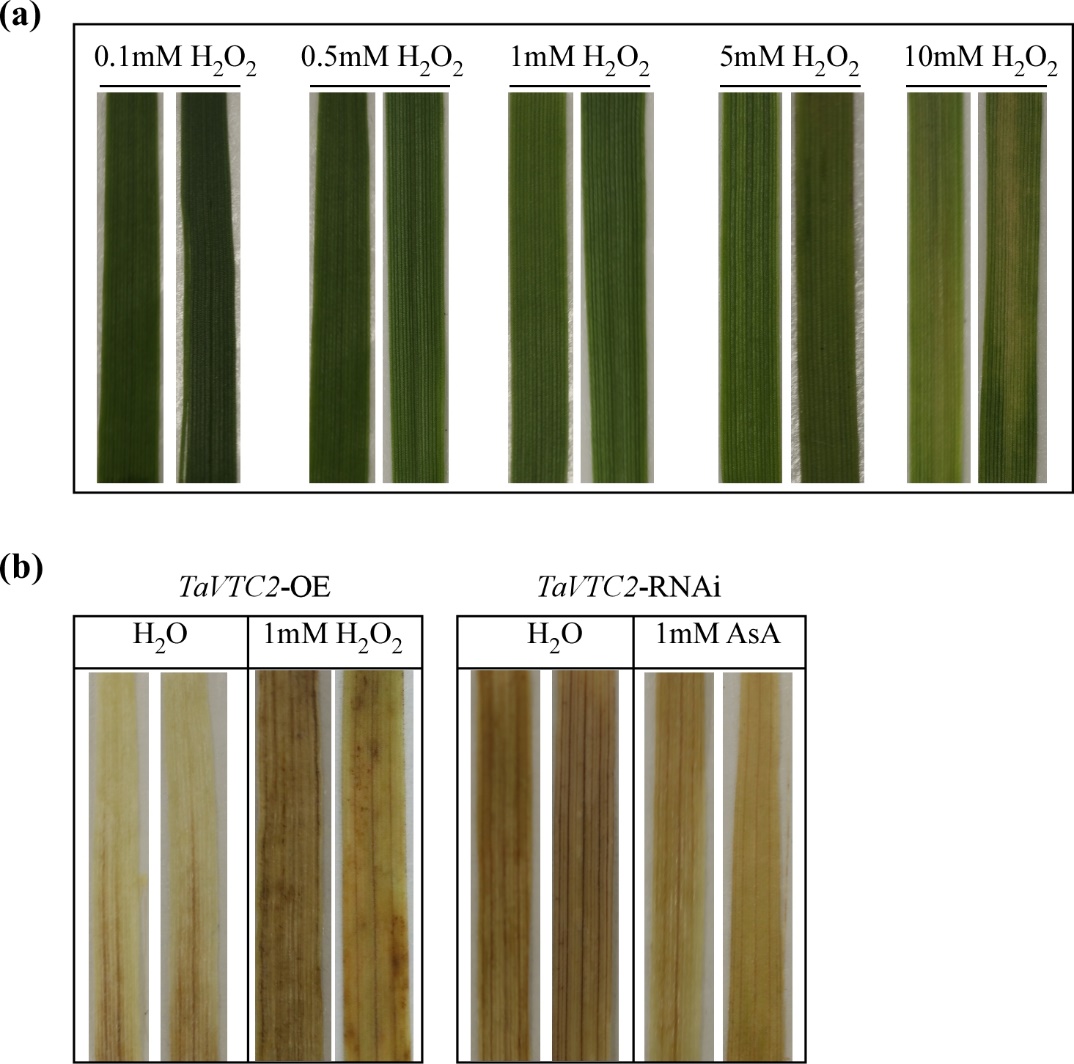


**Figure S5 Exogenous application of H_2_O_2_ to wheat plants and DAB staining. (a)** phenotype of wheat plants pretreated with different concentration gradients of H_2_O_2_ (0.1mM, 0.5 mM, 1 mM, 5 mM, 10 mM). (b) DAB staining of the leaves in *TaVTC2*-OE and *TaVTC2*-RNAi plant pretreated with 1mM H_2_O_2_ and 1mM AsA respectively, H_2_O was used as control.


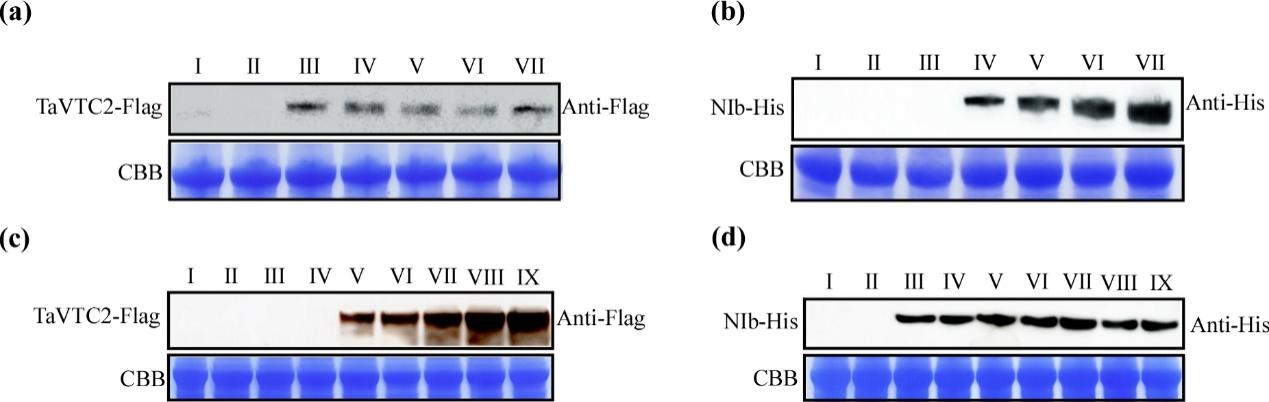


**Figure S6** **Western blot analysis of TaVTC2 protein and NIb protein expression.** (a) Western blot analysis of the TaVTC2-Flag accumulation in (Figure 6a) with anti-Flag antibody. (b) Western blot analysis of the NIb-His accumulation in (Figure 6a) with anti-His antibody. Ⅰ-Ⅶ represent MOCK, P.s.t. DC3000, P.s.t. DC3000 + 35S:TaVTC2-Flag (OD600=0.9), DC3000 + 35S:TaVTC2-Flag (OD600=0.9) + 35S:NIb-His (OD600=0.3), *P.s.t.* DC3000 + 35S:TaVTC2-Flag (OD600=0.9) + 35S:NIb-His (OD600=0.6), *P.s.t.* DC3000 + 35S:TaVTC2-Flag (OD600=0.9) + 35S:NIb-His (OD600=0.9), *P.s.t.* DC3000 + 35S:TaVTC2-Flag (OD600=0.9) + 35S:NIb-His (OD600=1.2). (c) Western blot analysis of the TaVTC2-Flag accumulation in (Figure 6d) with anti-Flag antibody. (d) Western blot analysis of the NIb-His accumulation in (Figure 6d) with anti-His antibody. Ⅰ-Ⅸ represent MOCK, *P.s.t.* DC3000, 35S:NIb-His, *P.s.t.* DC3000 + 35S:NIb-His (OD600=1), *P.s.t.* DC3000 + 35S:TaVTC2-Flag (OD600=0.4) + 35S:NIb-His (OD600=1), *P.s.t.* DC3000 + 35S:TaVTC2-Flag (OD600=0.8) + 35S:NIb-His (OD600=1), *P.s.t.* DC3000 + 35S:TaVTC2-Flag (OD600=1.2) + 35S:NIb-His (OD600=1), *P.s.t.*DC3000 + 35S:TaVTC2-Flag (OD600=1.6) + 35S:NIb-His (OD600=1), 35S:TaVTC2-Flag (OD600=2) + 35S:NIb-His (OD600=1). Coomassie brilliant blue (CBB) large stained rubisco gel was used to show protein loadings.


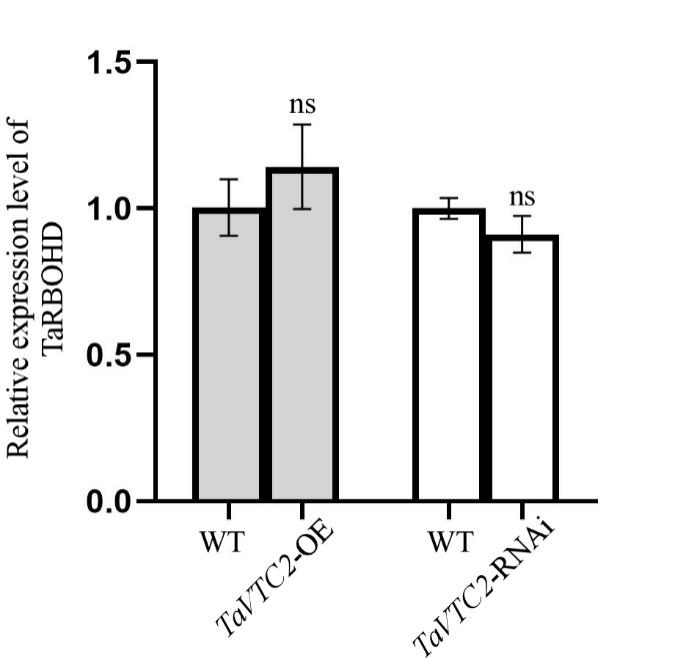


**Figure S7 Detection of *TaRBOHD* mRNA expression.** (a) Relative expression level of *TaRBOHD* in *TaVTC2*-OE/WT (field), *TaVTC2*-RNAi/WT (YM158) plants. Each relative expression level is presented as mean ± SD from three biological samples and each biological sample had three technical replicates. Statistical analyses were done using Student’s *t*-test. *, *P* < 0.05; **, *P* < 0.01; ns, no significant difference.


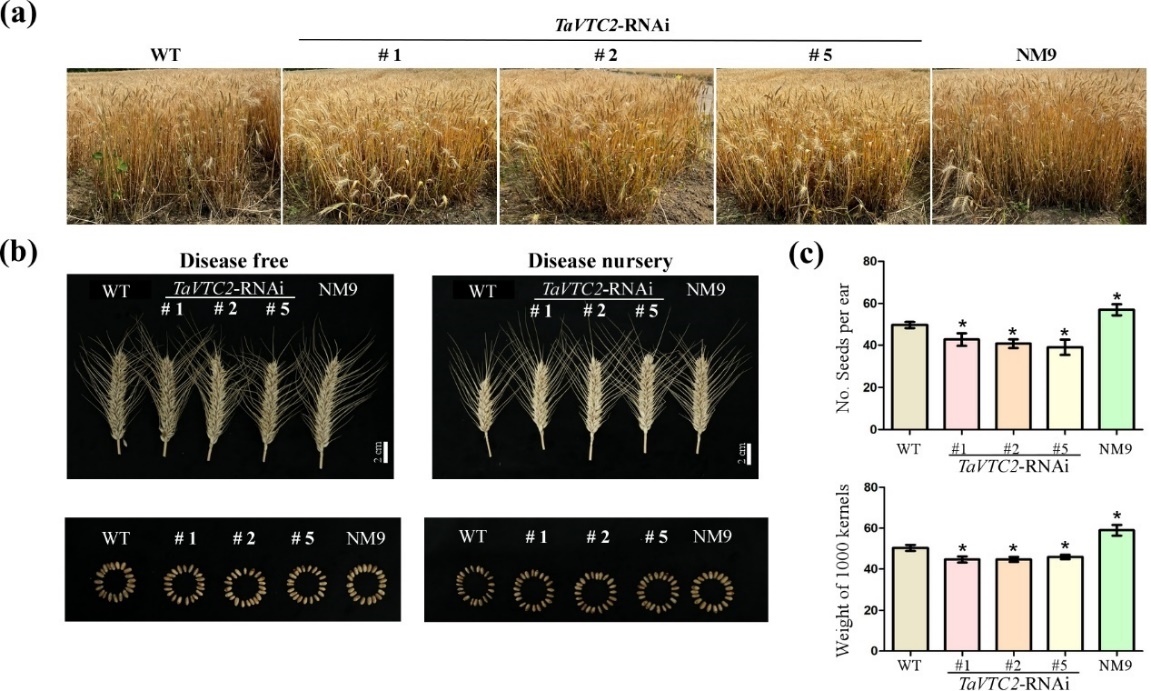


**Figure S8** **Field assessment of transgenic lines of *TaVTC2*-RNAi for agronomic traits.** (a) Field assay results showing *TaVTC2*-RNAi, YM158 (WT), and NM9 plants at maturity. Bar, 30 cm. (b) Comparison of seed quality and wheat ears among transgenic plants, NM9, and YM158 (WT) in the disease nursery or a disease-free field. Bar, 2.0 cm. (c) Analyses of seed number per wheat ear and 1000-kernel weight of the *TaVTC2*-RNAi, YM158 (WT), and NM9 plants at maturity.


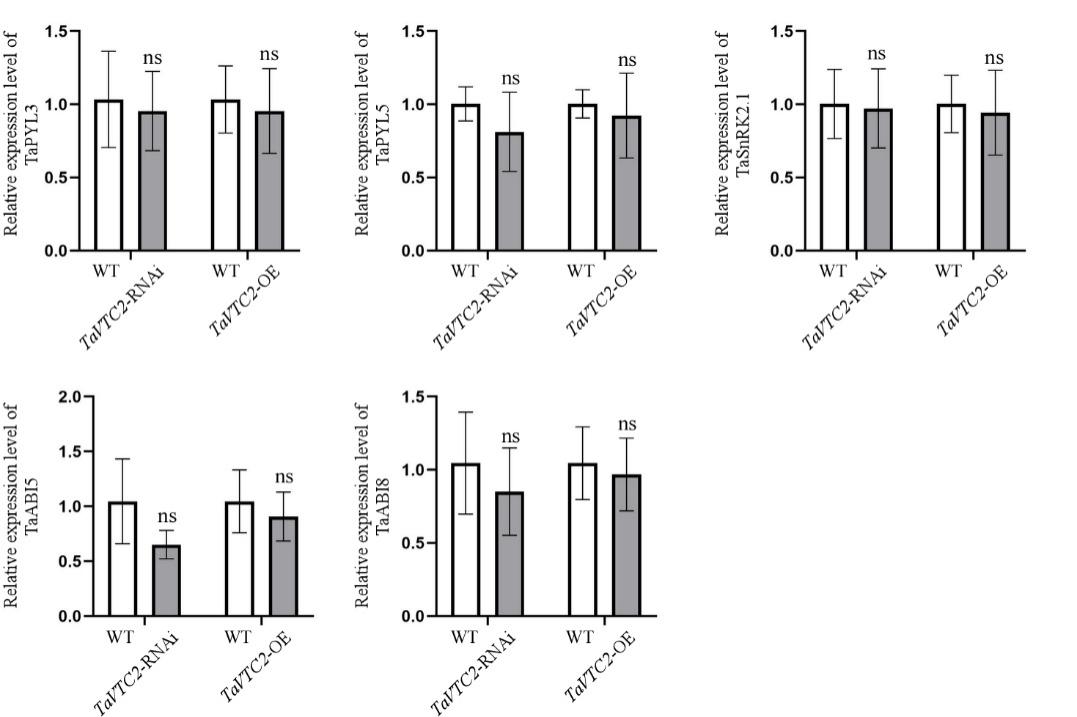


**Figure S9** **Relative mRNA expression level of ABA signaling pathway genes.** (a) Relative expression level of *TaPYL3, TaPYL5, TaSnRK2.1, TaABI5, TaABI8* in *TaVTC2*-OE/WT (field), *TaVTC2*-RNAi/WT (YM158) plants. Each relative expression level is presented as mean ± SD from three biological samples and each biological sample had three technical replicates. Statistical analyses were done using Student’s *t*-test. *, *P* < 0.05; **, *P* < 0.01; ns, no significant difference.
